# Design of a Trigger‐Responsive Photothermal Vesicle‐Based Cargo Delivery Platform

**DOI:** 10.1002/smll.202505852

**Published:** 2025-11-12

**Authors:** Anastassiya Schramm, Nina F. Conzelmann, Ann‐Kathrin Gelmroth, Julia Köberle, Stefan Schramm, Rumiana Dimova, Ilia Platzman, Joachim Spatz

**Affiliations:** ^1^ Department of Cellular Biophysics Max Planck Institute for Medical Research Jahnstraße 29 69120 Heidelberg Germany; ^2^ Max Planck School Matter to Life Jahnstraße 29 69120 Heidelberg Germany; ^3^ Organic Chemistry University of Applied Sciences Dresden Friedrich‐List‐Platz 1 01069 Dresden Germany; ^4^ Max Planck Institute of Colloids and Interfaces Science Park Golm 14476 Potsdam Germany; ^5^ Max Planck‐Bristol Center for Minimal Biology University of Bristol Bristol BS8 1TD UK; ^6^ Institute for Molecular Systems Engineering and Advanced Materials Heidelberg University Im Neuenheimer Feld 225 69120 Heidelberg Germany

**Keywords:** drug delivery, gold nanorods, GUVs, trigger‐responsive materials

## Abstract

Cargo delivery systems enable the targeted transport of therapeutic agents to specific sites, enhancing treatment efficacy while minimizing systemic side effects. However, many existing systems struggle with achieving precise control over the release timing. Here, a photothermal trigger‐responsive delivery platform based on Giant Unilamellar Vesicles (GUVs) functionalized with gold nanorods (GNRs) is described to overcome these challenges. Toward this end, cargo‐loaded GUVs are produced using the emulsion transfer method and effective GNR attachment to the vesicle membrane is achieved by functionalizing the GNRs with cholesterol. The near‐infrared light (NIR)‐mediated cargo release mechanism is described. Importantly, high release efficiency is achieved by optimizing the cholesterol concentration on the GNRs, which is essential for controlling the GNR attachment mechanism to the GUV membrane. Finally, GNR‐functionalized ampicillin‐loaded GUVs are tested in the presence of *E. coli* bacteria to demonstrate the platform's functionality. Following NIR‐triggered antibiotic release, bacterial growth is inhibited. This showcases the practical potential of the developed trigger‐responsive system. Notably, GNR‐mediated photothermal heating alone also reduces bacterial viability upon extended illumination, indicating a dual antibacterial mechanism. This versatile and biocompatible system offers a controlled delivery method with broad applicability for therapeutics, diagnostics, and other biomedical applications.

## Graphical abstract

Drug delivery is reimagined with a vesicle platform that responds only to light. Gold nanorods are anchored to giant unilamellar vesicles through cholesterol, and tuning the cholesterol concentration ensures optimal attachment and efficient release. Upon near‐infrared light stimulation, antibiotic cargo deploys on demand to inhibit bacterial growth, showcasing a programmable strategy for next‐generation therapeutics and biomedical technologies.

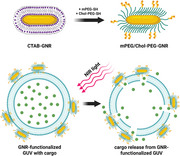

## 1 Introduction

Drug delivery systems play a crucial role in administering therapeutic agents by facilitating their transport to the target site in a controlled and efficient manner.^[^ ^1^, ^2^, ^3^ ^]^ These systems aim to enhance therapeutic efficacy, minimize side effects, and improve patient compliance.^[^ ^1^, ^4^, ^5^ ^]^ Delivery platforms, ranging from conventional systems (e.g., tablets, ointments, and capsules) to controlled delivery vehicles (e.g., stimuli‐responsive polymersomes, nanoparticles, and liposomes), offer distinct advantages as they are specifically tailored to various applications.^[^ ^6^ ^]^ In addition to their use in pharmaceutics, drug delivery systems have found broad applications in gene therapy,^[^ ^7^, ^8^, ^9^ ^]^ diagnostics,^[^ ^10^, ^11^ ^]^ and regenerative medicine,^[^ ^12^, ^13^ ^]^ demonstrating their versatility across biomedical fields. While these systems have revolutionized therapeutic delivery by improving bioavailability and stability, a key challenge remains: achieving precise control over drug release timing and location.

Regarding the targeting and release mechanisms, delivery systems can be classified into passive and active approaches. Passive delivery systems rely on spontaneous processes, such as enhanced permeability and retention (EPR) effects, which facilitate the accumulation of therapeutic agents at the target site. Drug release in these systems occurs when delivery particles fuse with or are internalized by target cells through cellular uptake mechanisms.^[^ ^14^ ^]^ While passive delivery systems are widely used and have shown potential,^[^ ^15^ ^]^ they are often associated with significant side effects, multidrug resistance, and a lack of specificity and control needed for optimal therapeutic outcomes.^[^ ^3^, ^16^ ^]^

To address these limitations, so‐called active or trigger‐responsive systems, have been developed. These advanced platforms enable controlled release and targeted delivery by responding to environmental or external stimuli.^[^ ^17^ ^]^ Internal triggers, such as pH,^[^ ^18^ ^]^ redox cues,^[^ ^19^ ^]^ oxygenation conditions,^[^ ^20^ ^]^ or enzymes^[^ ^21^ ^]^ leverage natural physiological variations at the target site to achieve localized release. However, these systems are inherently constrained by their reliance on local conditions, reducing the precision and external control over the delivery process. In contrast, externally triggered systems provide a more robust solution by allowing precise temporal and spatial control over cargo release. These systems can be activated by external stimuli, such as ultrasound,^[^ ^22^ ^]^ magnetic field,^[^ ^23^ ^]^ or light,^[^ ^24^ ^]^ thus enabling tailored intensity and improved therapeutic efficacy. Among these, ultrasound and magnetic field‐based systems have demonstrated utility but continue to face challenges, such as limited penetration, potential tissue and DNA damage, inaccurate targeting, and high equipment costs. These challenges make them less practical for certain biomedical applications.^[^ ^25^, ^26^ ^]^

Among externally triggered delivery systems, light‐activated platforms stand out for their noninvasive nature, precise controllability, and adaptability to diverse therapeutic applications. These systems provide spatial and temporal control over the delivery process, enabling more effective treatment strategies.^[^ ^27^, ^28^ ^]^ While UV light is often used for such systems,^[^ ^29^ ^]^ it poses some phototoxic risks. Near‐infrared (NIR) light serves as a safer, noninvasive, remote‐controlled alternative with the advantages of deep tissue penetration, minimal scattering, and reduced photodamage.^[^ ^28^ ^]^ Gold nanoparticles (GNPs), and particularly gold nanorods (GNRs), are well‐suited for these systems due to their tunable surface plasmon resonance in the NIR region, enabling efficient photothermal conversion and controlled drug release.^[^ ^30^, ^31^, ^32^ ^]^ Unlike other GNPs, GNRs inherently offer superior tunability in the NIR range and efficiency in photothermal applications without requiring large sizes or complex modifications.^[^ ^33^, ^34^ ^]^ When combined with carriers like droplets or liposomes, GNRs facilitate multifunctional delivery systems capable of controlled drug release, on‐demand responsiveness, and multimodal imaging and therapy.^[^ ^35^, ^36^, ^37^, ^38^ ^]^

Among various carriers for delivery systems, liposomes have gathered significant attention as transport systems in the food, cosmetic, and pharmaceutical industries. Particularly, Large Unilamellar Vesicles (LUVs), with their biocompatibility and ability to improve pharmacokinetics and pharmacodynamics have been extensively used as delivery agents.^[^ ^39^, ^40^ ^]^ Despite their wide use, their small size (≈100 nm) imposes limitations, including low encapsulation efficiency and constraints on the size of the cargo. To address these challenges, Giant Unilamellar Vesicles (GUVs) have emerged as a promising alternative.^[^ ^41^ ^]^ With radii exceeding 1 µm, GUVs can encapsulate larger quantities of active compounds and accommodate diverse cargo types.^[^ ^39^, ^42^ ^]^ Additionally, their micrometer‐scale size allows for primary targeting, enabling specific accumulation of therapeutics in desired organs.^[^ ^43^ ^]^ Furthermore, their larger size enhances microscopy observations, enabling the characterization of membrane mechanical properties and a detailed investigation of interaction mechanisms.^[^ ^44^, ^45^, ^46^ ^]^ GUVs also offer versatility in modulating cell‐carrier interactions through the incorporation of ligands or repellant motifs, improving fusogenicity and internalization. Studies have demonstrated that micrometer‐scale liposomes can target specific organs, undergo cellular internalization, and interact with intracellular constituents, further highlighting their potential as advanced cargo carriers.^[^ ^43^ ^]^

To this end, multiple studies have explored GNP‐liposome hybrid systems for controlled drug release, achieving various degrees of success. A common feature of many such systems is their reliance on thermosensitive lipids, such as lysolipids, 1,2‐dipalmitoyl‐sn‐glycero‐3‐phosphocholine (DPPC), and 1,2‐distearoyl‐sn‐glycero‐3‐phosphocholine (DSPC),^[^ ^47^, ^48^, ^49^, ^50^ ^]^ which exhibit transition temperatures between 40 and 60 °C. While these lipids enable temperature‐responsive release, they are prone to premature leakage of drugs at physiological temperatures,^[^ ^47^, ^51^, ^52^ ^]^ which limits their reliability. Another challenge lies in the use of cationic lipids to enhance GUV functionality.^[^ ^53^ ^]^ Although effective, these lipids can be toxic to cells, raising biocompatibility concerns.^[^ ^54^ ^]^ Beyond lipid composition, the method of attaching GNPs to liposomes presents additional limitations. In some systems, GNPs are grown directly on the liposomal surface using strong reducing agents, which risk damaging the vesicle structure.^[^ ^53^ ^]^ Alternatively, encapsulating GNPs within liposomes has been shown to facilitate cargo release, but this can also degrade sensitive encapsulated compounds, such as proteins, due to localized heating.^[^ ^49^ ^]^ The type of gold nanoparticles used also influences system performance. For instance, spherical nanoparticles,^[^ ^55^, ^56^, ^57^ ^]^ hollow gold nanoshells,^[^ ^49^ ^]^ and nanostars^[^ ^58^, ^59^ ^]^ have been employed in various designs. However, their activation relies either on UV light,^[^ ^60^ ^]^ which poses phototoxicity concerns, or requires a pulsed laser,^[^ ^47^, ^49^, ^58^ ^]^ which adds complexity, cost, and high energy demands to the release process. These limitations collectively underscore the need for improved hybrid systems that combine biocompatibility, stability, and precise control over drug release.

This research addresses the abovementioned challenges by presenting a modular lipid vesicle‐based delivery platform, designed for NIR light‐triggered release. The platform combines GUVs as the carrier compartment, capable of encapsulating a wide variety of cargo types, and GNRs as the trigger‐responsive element, offering efficient photothermal conversion properties for precise cargo release upon NIR illumination. This system utilizes stable, biocompatible lipid compositions to ensure reliability under physiological conditions. The system also employs a cost‐effective NIR light‐emitting diode (LED) setup instead of pulsed laser, and positions GNRs on the outer surface of GUVs to minimize cargo damage. Designed for stability, biocompatibility, and versatility, it can encapsulate a wide variety of substances while responding efficiently to an external trigger. By leveraging the unique properties of GNRs and lipid vesicles, this platform offers a promising foundation for trigger‐responsive therapeutic and diagnostic applications, with the added benefit of being more accessible and user‐friendly than conventional laser‐based systems.

## 2 Results and Discussion

The light trigger‐responsive system consists of two main modules: 1) the GUV‐based cargo‐loaded compartment; and 2) the NIR light‐triggered release module achieved by functionalization of the outer side of the GUV's membrane with GNRs. GUV‐based compartments have been selected due to their potential advantages as a cargo delivery system. External attachment of GNRs was employed to minimize the potential NIR‐induced heat damage to the encapsulated cargo.

### 2.1 Preparation and Surface Modification of Gold Nanorods

**Figure 1:**
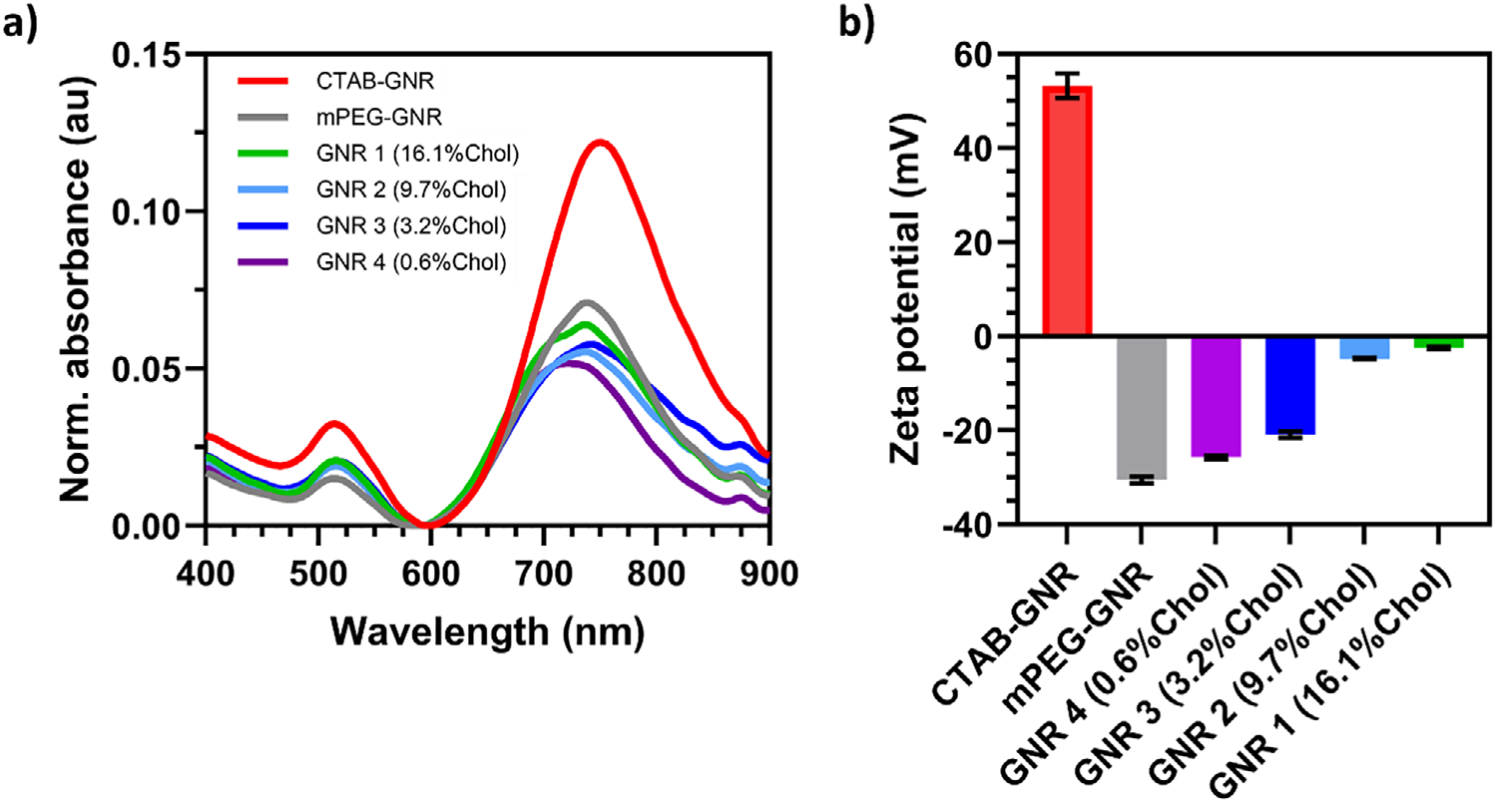
Characterization of functionalized GNRs. a) Absorbance spectra, and b) zeta potential of GNRs with different coatings: CTAB‐GNR; mPEG‐GNR; GNR 1 (16.1% Chol), GNR 2 (9.7% Chol), GNR 3 (3.2% Chol), and GNR 4 (0.6% Chol). *N* = 3 for zeta potential measurements.

GNRs were synthesized via a silver‐assisted, seed‐mediated growth method (see the Experimental Section).^[^ ^61^ ^]^ The synthesis was optimized to produce GNRs with an average length of 28 nm and a diameter of 9 nm, as determined from the scanning electron microscopy (SEM) analysis (Figure S1, Supporting Information). Localized surface plasmon resonance (LSPR) bands at 512 nm (transverse) and 753 nm (longitudinal) (**Figure** **1**a) along with a zeta potential of 53.2 ± 2.6 mV confirmed successful GNR synthesis (Figure 1b).^[^ ^28^, ^62^ ^]^ The obtained GNR dimensions and aspect ratio between 3 and 4 are essential for efficient light‐to‐heat conversion while minimizing the deformation of lipid vesicles during conjugation.^[^ ^61^, ^63^ ^]^

To adapt the produced GNRs for biological applications, we set out to replace the nanoparticle coating from highly cytotoxic^[^ ^64^ ^]^ Cetrimonium bromide (CTAB) to a biocompatible polyethylene glycol (PEG).^[^ ^65^, ^66^ ^]^ CTAB was effectively replaced by mPEG(2000)‐SH in TRIS buffer at low pH, following an optimized protocol from Zhang et al.^[^ ^67^ ^]^ The resulting GNRs are referred to as mPEG‐GNRs (see the Experimental Section). Zeta potential measurements revealed a change in the charge of GNRs from highly positive 53.2 ± 2.6 mV for the CTAB‐GNRs to negative ‐30.5 ± 0.7 mV for the mPEG‐GNR, thus confirming successful GNR‐surface coating with PEG (Figure 1b).^[^ ^68^, ^69^ ^]^

In addition to coating the GNRs with PEG, we decided to implement cholesterol‐functionalized PEG thiol (Chol‐PEG(2000)‐SH) to enable the integration of GNRs into the outer leaflet of the lipid vesicles. We selected Chol‐PEG(2000)‐SH as the membrane‐tethering ligand because cholesterol functionalization of nanoparticles/liposomes has been shown to enhance in vivo stability and prolong circulation in serum‐rich environments, thereby providing a robust anchor at physiological interfaces.^[^ ^70^, ^71^ ^]^ Given cholesterol's impact on membrane organization,^[^ ^72^ ^]^ we systematically varied the surface density of Chol‐PEG(2000)‐SH on the GNRs to tune cholesterol content and thereby control binding to GUVs and release. Toward this end, GNRs were functionalized with various ratios of mPEG(2000)‐SH to Chol‐PEG(2000)‐SH (see Table S1, Supporting Information). The following four GNRs were obtained (the percentages given in the brackets refer to the relative amounts of cholesterol‐functionalized PEG added during functionalization): GNR 1 (16.1% Chol); GNR 2 (9.7% Chol); GNR 3 (3.2% Chol); and GNR 4 (0.6% Chol). As a clarification, in this study we treat the mPEG‐SH/Chol‐PEG‐SH shell as ensemble‐averaged and report nominal cholesterol feed ratios from the ligand‐exchange step without assigning tip—sidewall differences. Both mPEG‐GNRs and mPEG/Chol‐PEG‐functionalized GNRs exhibited blueshift in LSPR of ≈16 nm due to the change in the refractive indexes from CTAB (1.46) to mPEG (1.40)^[^ ^73^ ^]^ (Figure 1a). Within this ensemble view, the addition of cholesterol altered dispersion behavior: over time, GNR sedimentation was observed, consistent with increased shell hydrophobicity and weak, reversible interparticle association. Importantly, samples readily redispersed with gentle pipetting or sonication, indicating reversible clustering rather than irreversible aggregation. While curvature‐biased enrichment of cholesterol could modulate local interaction strength, such effects would average across the population and are not expected to impact the functional outcomes reported here.

Zeta potential measurements of cholesterol‐functionalized GNRs revealed a decrease in the negative charge with increasing cholesterol content (Figure 1b). This trend can be attributed to the cholesterol's propensity to self‐stack due to its hydrophobic nature, leading to the formation of aggregated cholesterol domains.^[^ ^74^ ^]^ These domains, in turn, facilitate a more ordered arrangement of PEG molecules on the GNR surface, enhancing steric shielding and effectively dampening the observed negative charge.

### 2.2 Preparation of Single‐Compartment and Multicompartment GUVs

**Figure 2:**
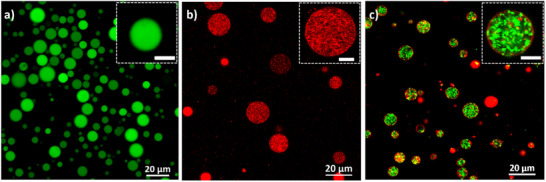
Cargo‐loaded GUV analysis. GUVs containing a) calcein (green signal), b) 100 nm SUVs (red signal: LissRhod PE in SUV membrane); and c) 200 nm fluorescent beads (green signal: beads; red signal: LissRhod PE in GUV membrane). The insets in the top right corner of each image show a magnified view of a single GUV from each sample. Scale bars in the insets are 5 µm.

GUVs were chosen as cell‐sized cargo carriers due to their ability to encapsulate a wide range of cargo types, making them ideal for diverse delivery applications.^[^ ^39^ ^]^ For GUV formation we chose the emulsion transfer method (see the Experimental Section).^[^ ^75^ ^]^ This method is easily adaptable for the encapsulation of various small cargos, such as calcein and ampicillin, as well as larger cargos, such as 100 nm‐sized SUVs and 200 nm fluorescent beads (**Figure** **2**). Unless otherwise specified, all GUVs were composed of 1,2‐dioleoyl‐sn‐glycero‐3‐phosphocholine (DOPC) to 2‐dioleoyl‐sn‐glycero‐3‐ phospho‐(1′‐rac‐glycerol) (sodium salt) (DOPG) lipids mixed at a 4:1 molar ratio. In contrast to lipids like DPPC, which remain in the gel phase at room temperature and therefore require vesicle preparation at elevated temperatures, DOPC exists in the fluid phase under ambient conditions, eliminating the need for heating and simplifying the production process. The addition of small amounts of DOPG allows for the formation of slightly negative biocompatible vesicles. Moreover, negatively charged DOPG enhances the binding and uptake of vesicles by cells, which is essential for efficient therapeutic delivery.^[^ ^39^, ^76^ ^]^ It is important to mention here that in all emulsion transfer preparations the interior and exterior buffers of the GUVs were carefully matched in osmolarity to ensure consistency across samples with different encapsulated cargos.

Figure 2 shows representative fluorescence images of GUVs loaded with calcein, SUVs, or fluorescent polystyrene beads. Unlike microfluidics‐based methods, which typically produce more uniform vesicles, this approach results in a heterogeneous population. GUVs loaded with calcein exhibit the largest size distribution, ranging from 3 to 30 µm, with an average vesicle diameter of ≈11 µm (Figure S3, Supporting Information). SUV‐loaded GUVs display a similarly broad size range (1–25 µm) and a similar average diameter (≈10 µm). Interestingly, fluorescent bead‐loaded GUVs exhibited the smallest size distribution, with diameters ranging from 2 to 14 µm and an average of ≈7 µm. The reduced size distribution and diameter of bead‐loaded GUVs can be attributed to the rigid and large nature of the encapsulated cargo. The rigidity and the size of the beads may induce elevated membrane tension during centrifugation cycles, leading to the generation of smaller GUVs. To ensure accurate size estimation, vesicles were imaged using both fluorescence and transmitted light channels. Also, measurements were only taken from GUVs whose equatorial planes were clearly in focus and whose membrane outlines matched in both channels. This approach minimized potential artifacts from partial cross‐sections and ensured consistency across all vesicle types. Importantly, no interactions between the cargo and the GUV membranes were observed, as evidenced by the absence of colocalization between the membrane signal and the fluorescence signal from the cargo. This result is consistent with expectations, as charge‐mediated repulsion between the negatively charged beads, calcein and DOPG‐containing SUVs as well as the negatively charged GUV membranes is expected. Calcein‐ and SUV‐loaded vesicles show uniform distribution of the cargo within the lumen of the GUVs. In contrast, the distribution of the cargo in the bead‐loaded GUVs is less uniform due to transient dynamic association of the beads in the buffer. The speckled texture in Figure 2b,c reflects motile submicrometer cargo (encapsulated SUVs and carboxylate polystyrene beads), whose Brownian motion produces blurred intensity in a single focal plane. Under the low‐ionic‐strength, divalent‐cation‐free buffer conditions used here, sustained aggregation is disfavored, and we did not detect stable clusters.

The emulsion transfer method for the formation of cargo‐loaded GUVs is simple and adaptable. Unlike microfluidics or other controlled production techniques it does not require specialized equipment, making it a highly versatile approach for encapsulating diverse cargos in an easy and efficient way. Although other methods achieve more consistent size uniformity, the approach we chose was sufficient to demonstrate the system's potential for cargo release experiments. However, it is important to mention here that the encapsulation efficiency was estimated to be around 3% (see the Experimental Section; and the Supporting Information) which might be lower than what has previously been achieved utilizing a microfluidic approach.^[^ ^77^ ^]^ In future iterations, higher loading could be achieved via continuous‐flow microfluidics (e.g., octanol‐assisted liposome assembly (OLA) or double‐emulsion generators) or ontinuous droplet interface crossing encapsulation (cDICE) to improve control over vesicle size and encapsulation. Shifting dosing studies to LUVs that permit active loading and high drug‐to‐lipid ratios, as well as postformation enrichment through PEG‐ or Ca^2^⁺‐mediated vesicle fusion could further boost intravesicular cargo without altering the trigger chemistry.^[^ ^78^, ^79^ ^]^

### 2.3 Functionalization of GUVs with GNRs

To assess the binding of cholesterol‐functionalized GNRs to the cargo‐loaded GUVs, we decided to add a fluorophore‐containing linker FITC (fluorescein isothiocyanate)‐PEG(5000)‐SH to the GNR coating. Note, the PEG spacer of 5000 Da was chosen to prevent fluorescence quenching.^[^ ^80^ ^]^ The concentration of FITC‐PEG(5000)‐SH was kept below 5% mol to ensure a sufficient fluorescence signal for GNR visualization while minimizing its interference in the coating process. Imaging experiments were performed using GNRs functionalized with 10.6% Chol‐PEG due to a slight variation in the calculated mixing ratio; this composition is close to the 9.7% formulation and falls within the range of tested conditions. For assessing the binding of GNRs to GUVs (see the Experimental Section) the GNRs without cholesterol were implemented as a control.

**Figure 3:**
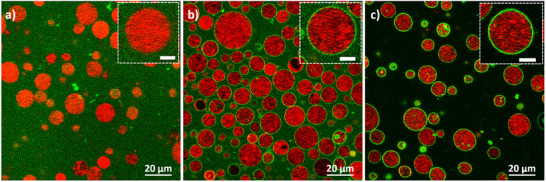
Assessing the effect of GNR‐functionalization on the interaction of GNRs with SUV‐loaded GUVs. Representative confocal cross‐section images of SUV‐loaded GUVs incubated with: a) FITC‐functionalized GNRs without a cholesterol coating, b) FITC‐functionalized cholesterol‐coated GNRs, or c) FITC‐functionalized cholesterol‐coated GNRs after the removal of unbound GNRs. Red and green signals correspond to LissRhod PE on SUVs and FITC‐coated GNRs, respectively. The insets in the top right corner of each image show a magnified view of a single GUV from each sample. Scale bars in the insets are 5 µm.

FITC‐labeled GNRs were added to the SUV‐loaded GUVs and gently mixed. In the control sample GNRs remained dispersed in the solution and the GUVs were clearly visible due to the encapsulated SUVs (**Figure** **3**a). In the magnified image it is evident that the GNRs do not attach to the GUV membrane. In the case of cholesterol‐functionalized GNRs, a green fluorescent signal on the GUVs membranes is observed, indicating successful incorporation of the GNRs into the membranes of the vesicles (Figure 3b).

It is crucial to minimize nonspecific heating by the unbound GNRs and to ensure biocompatibility of the GNRs‐functionalized GUVs for potential biomedical applications. To address this, unbound GNRs were removed by centrifugation at 380 g. The postpurification sample (Figure 3c) shows reduced background signals stemming from unbound FITC‐functionalized GNRs, while the fluorescence intensity at the GUV periphery remains unchanged. This confirms that attached GNRs were unaffected by purification. To ensure biocompatibility, cell viability tests were performed using fibroblast cells exposed to CTAB‐GNRs or PEG‐GNRs as well as nonfunctionalized and GNR‐functionalized GUVs. As expected, CTAB‐GNRs were highly cytotoxic,^[^ ^64^ ^]^ whereas the other three (PEG‐GNRs, nonfunctionalized and GNR‐functionalized GUVs) showed nearly 100% viability (Figure S4, Supporting Information). These results indicate that the potentially remaining CTAB concentration on the GNRs is below its cytotoxicity level.

### 2.4 Release of the Cargo from the GNR‐Functionalized GUVs

After developing biocompatible and stable GNR‐functionalized GUVs, we set out to evaluate their cargo release capability by illuminating the samples with NIR light. Toward this end, an NIR LED illumination system (730 nm) was designed (see Figure S5, Supporting Information). The system provided a maximum power density of 4 W cm^−^^2^ under continuous‐wave (CW) illumination, and the calibration curve (Figure S6, Supporting Information) ensured optimal radiometric LED power.

Based on the hypothesis that higher cholesterol content might promote better GNR attachment to the GUV surface we set out to investigate calcein release using GNRs containing 16.1% cholesterol. Calcein was chosen as a fluorescent marker because it is commonly used for release studies.^[^ ^81^ ^]^ Release efficiency was calculated as the ratio of calcein concentration in supernatant to the total calcein content (inside GUVs + supernatant), expressed as a percentage (see the Experimental Section). While inhomogeneous encapsulation of calcein across individual GUVs is an inherent limitation of the emulsion transfer method, our analysis is based on bulk measurements of the entire GUV population. As such, individual variability is averaged out and does not significantly impact the interpretation of release efficiency on the population level. Moreover, due to the nature of this GUV production technique, controlling or quantifying encapsulation homogeneity at the single‐vesicle level is not feasible within the scope of this study.

Surprisingly, relatively low release efficiencies of 12.7% ± 0.7%, 14.5% ± 1.3%, and 24.5% ± 2.9% were achieved after 1, 5, and 10 min, respectively (Figure S7, Supporting Information). Longer illumination times were not tested, as extended NIR exposure at a power density of 4 W cm^−^^2^ could lead to excessive heating, potentially causing hyperthermia in biological tissues, which is a critical concern in biomedical applications (Figure S10b, Supporting Information). Note, calcein‐loaded nonfunctionalized GUVs that have been illuminated with NIR showed minimal (<5%) spontaneous leakage similar to nonilluminated samples (Figure S7a,b, Supporting Information). This leakage can be attributed to natural membrane fluctuations or handling during centrifugation and pipetting.^[^ ^82^ ^]^ Nonilluminated GNR‐functionalized GUVs showed slightly higher baseline leakage levels of ≈15% (for 10 min). The increased membrane permeability of the GNR‐functionalized GUVs can be explained by the mismatch between the lipids in the inner and outer membrane leaflets due to GNRs insertion. Excessive cholesterol from the GNRs can cause an asymmetric membrane, and hence, leaflet stress.^[^ ^83^ ^]^ The relatively low release efficiency as obtained with GNRs containing a high cholesterol concentration (16.1%) can be attributed to a premature bulky aggregation of GNRs, leading to unstable attachment to the membrane and, as consequence, their loss during the purification step. Therefore, we set out to test the release efficiency with GNRs containing reduced cholesterol content, hypothesizing that lower levels may minimize premature aggregation and promote more favorable particle dispersion and membrane binding.

**Figure 4:**
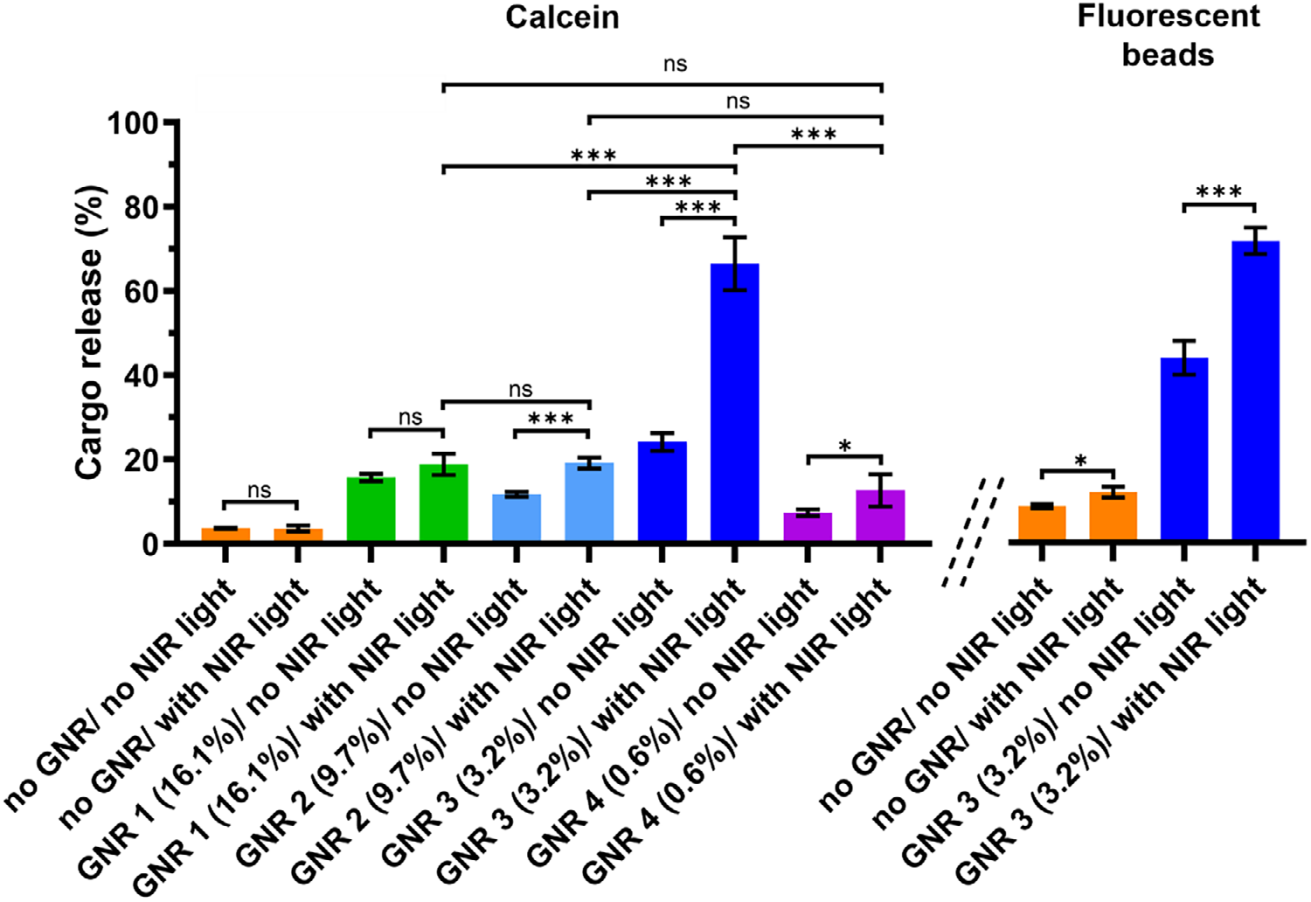
Cargo release efficiency from GUVs after 10 min of NIR light illumination (CW NIR LED, 4 W cm^−^^2^). Release from calcein‐loaded GUVs is shown for GNR formulations 1–4; release from fluorescent polystyrene bead‐loaded GUVs is shown for GNR 3 only. Data are mean ± SD; *N* denote independent GUV preparations (separate vesicle batches). Controls include GUVs without GNRs and matched no‐illumination conditions for each cargo. Sample sizes: calcein—controls (no GNRs), *N* = 3; with GNRs, *N* = 5 each. Beads—controls (no GNRs), *N* = 4; with GNRs, *N* = 5. Statistics: Tests and multiple‐comparison handling are described in the Experimental Section, Statistical tests. Exact *p*‐values (Calcein, illuminated vs nonilluminated): control (no GNRs): 0.724; GNR 1: 0.208; GNR 2: 3.74×10^−4^; G NR 3: 5.60×10^−4^; GNR 4: 0.011. Between‐formulation (Calcein, illuminated) pairwise *p*‐values are provided in Table S2 (Supporting Information) (e.g., GNR 1 vs GNR 3: 6.10×10^−4^; GNR 2 vs GNR 3: 4.66×10^−6^). Beads *p*‐values (illuminated vs nonilluminated): control (no GNRs): 7.21×10^−6^; GNR 3: 0.0144.

**Figure** **4** shows calcein release efficiency from GUVs functionalized with GNRs containing 16.1%, 9.7%, 3.2%, or 0.6% cholesterol. As can be observed, reduced release efficiency was a consequence of both high and low cholesterol content. These results support our earlier hypothesis that GNR 4 (0.6% Chol) likely lacked sufficient cholesterol for effective GUV attachment. Those with high cholesterol content, GNR 1 (16.1% Chol) and GNR 2 (9.7% Chol), on the other hand, induce GNR aggregation, which ultimately affects efficient binding to the GUV membranes. In contrast, GNR 3 (3.2% Chol) demonstrates the highest release efficiency, reaching 66.5% ± 6.3%. Importantly, absolute release efficiencies can be influenced by passive leakage, handling‐related shear, and vesicle size heterogeneity; we therefore interpret outcomes relative to matched controls processed in parallel. While some degree of GNR aggregation is still expected at this cholesterol content (and also evident from the sample sedimentation over time), this aggregation may in fact be beneficial. When controlled, nanoscale clustering of GNRs can enhance photothermal efficiency through plasmonic coupling, as long as the aggregates remain small enough to avoid the adverse effects associated with excessive aggregation.^[^ ^84^ ^]^ These findings highlight the importance of the cholesterol‐to‐PEG ratio in regulating cargo release. Important to mention here is that the effective release of around 70% was achieved with low power density (4 W cm^−^^2^) illumination, which is lower than what is used in similar systems (7–18 W cm^−^^2^).^[^ ^82^, ^85^ ^]^ Moreover, some hybrid systems were not able to achieve effective release under CW lasers due to insufficient temperature gradients.^[^ ^86^, ^87^ ^]^

To evaluate the release efficiency of a larger cargo, fluorescent polystyrene beads (0.2 µm) were incorporated into the GUVs using the same emulsion transfer method and GUVs were functionalized with GNR 3 (3.2% Chol). The release efficiency for polystyrene beads was similar to that for calcein release: after NIR illumination a release efficiency of 71.2% ± 3.1% was reached (Figure 4). However, bead‐loaded GUVs exhibited higher spontaneous leakage in the absence of NIR illumination. Consistent with results above, this spontaneous leakage can be attributed to the rigidity and the size of the beads that induce elevated membrane tension during release assay centrifugation cycles. Despite these challenges, the GNR‐GUV system demonstrated efficient release of larger cargo under NIR illumination, highlighting its adaptability for diverse payloads. Optimizing GNR concentrations or refining purification protocols may further enhance retention of larger cargo.

Beyond the effect of heat generation on cargo release capabilities, the incorporation of GNRs into the GUV membrane also affects biophysical properties of lipid membranes that, in turn, can mediate spontaneous leakage and/or enhance NIR‐triggered cargo release. To investigate this aspect, we compared the bending rigidity of GNR 3‐functionalized GUVs to the nonfunctionalized GUVs. GNR 3‐functionalized GUVs were chosen for this analysis as they had the highest release efficiency and spontaneous leakage. The bending rigidity results revealed lower values for nonfunctionalized (15.6 ± 3.5 *k*_B_ *T*) in comparison to GNR 3‐functionalized GUVs (21.7 ± 4.8 *k*_B_ *T*). It is known that cholesterol incorporation does not significantly affect the bending rigidity of GUVs with unsaturated lipids like DOPC.^[^ ^88^, ^89^, ^90^ ^]^ Therefore, the observed mild increase in bending rigidity can be attributed to the adsorption of GNRs. These likely act as rigid inclusions, limiting membrane flexibility and capacity to deform.^[^ ^91^ ^]^ This interpretation is also consistent with previous reports of membrane stiffening (and even rigidification and pore formation) induced by the adsorption of silica nanoparticles.^[^ ^92^ ^]^ Overall, these findings suggest that GNR incorporation modulates membrane mechanics and contributes to better cargo release.

### 2.5 GNR‐Mediated Release Mechanism

In the literature, cargo release from GNR‐surrounded/loaded/functionalized GUVs is attributed to various mechanisms. These include lipid membrane phase transition and transient pore formation due to localized heating of nanorods as well as vesicle bursting due to osmotic stress.^[^ ^82^, ^93^, ^94^, ^95^ ^]^ In our GNR‐functionalized GUVs the release is unlikely to be attributed to lipid phase transitions, because of the low transition temperatures of DOPC and DOPG (≈‐20 °C)^[^ ^96^, ^97^ ^]^ lipids.^[^ ^98^ ^]^ Bursting of the vesicles is also not a plausible mechanism, since the GUVs can still be observed as intact following their illumination with NIR light (Figure S9, Supporting Information). Therefore, the most probable release mechanism involves GNR‐mediated heating in close proximity to the membrane, leading to vapor bubble formation and, as a consequence, to the generation of transient pores. While these transient bubbles are typically generated by short laser pulses (nano‐ to picosecond),^[^ ^93^ ^]^ they can also form under CW laser illumination when local temperatures reach ≈200 °C.^[^ ^99^ ^]^ In this work, the implemented LED system (4 W cm^−2^) and the applied GNRs have the potential to reach local temperatures that have been shown to cause nanobubble formation.^[^ ^100^ ^]^ Consequently, we hypothesize that the primary release mechanism involves temporary membrane destabilization driven by localized heating in close proximity to the membranes.

To validate this hypothesis and explore factors contributing to variations in release efficiency among GNRs coated with different cholesterol concentrations, we investigated the heating rates of the standalone GNRs in water. Temperature measurements were performed using a thermocouple inserted into the sample solution. For these experiments, GNR suspensions were kept in open tubes and illuminated with NIR light; the temperature was recorded every 10 s for the first 2 min and every 1 min thereafter. Temperature measurements revealed that GNRs 3 and 4 (i.e., those produced with 3.2% and 0.6% Chol) exhibited the highest heating rates, reaching 70 °C within 10 min. This is similar to what was measured with CTAB‐GNRs (Figure S10a, Supporting Information). In contrast, GNRs 1 and 2 reached ≈40 °C within 10 min. These findings indicate that high cholesterol‐mediated aggregation of GNRs (i.e., above 3.2%) negatively affects the heating capacity and, in consequence, the release efficiency. Note that the reported temperatures are bulk values; under NIR irradiation, GNRs generate localized interfacial hot zones at membrane contacts. It is this photothermal localization—not uniform bulk heating—that serves as the operative trigger of transient permeability. Accordingly, reproducing the same bulk temperature by conventional heating is not expected to mimic the light‐triggered release in the absence of localized nanoscale heating.

We further hypothesized that the attachment of GNRs to the GUV membrane also plays a significant role in the release process. To test this, we measured the temperature behavior of GNR‐functionalized GUVs under NIR illumination (Figure S10b, Supporting Information). GUVs were illuminated for defined time points, then immediately measured using a thermocouple, a procedure similar to the experiments with standalone GNRs. GUVs functionalized with GNR 1 (16.1% Chol), GNR 2 (9.7% Chol), and GNR 4 (0.6% Chol) exhibited a modest temperature increase of ≈15 °C, reaching only 45 °C after 10 min of illumination. In contrast, NIR illumination of GNR 3 (3.2% Chol)‐functionalized GUVs led to a faster and higher heating rate, reaching 70 °C within 6 min. These results can be attributed to differences in cholesterol content and membrane attachment stability, which in the case of 3.2% cholesterol resulted in the formation of small‐sized aggregates. GNRs that link with each other are known to exhibit distinct optical properties that differ to those of individual particles. The reason behind this is increased plasmonic coupling between closely packed GNRs, which enhances localized electromagnetic fields. This, in turn, leads to greater heat generation and improved release efficiency.^[^ ^101^, ^102^, ^103^, ^104^ ^]^ In the case of high cholesterol content (i.e., 16.1% and 9.7% cholesterol content during production) large aggregates might form. Two possible consequences of this are unstable attachment to the GUVs—leaving only a small number of individual GNRs on the membranes—or attachment of large aggregates that nonetheless exhibit reduced photothermal efficiency due to impaired heat generation.^[^ ^105^ ^]^ In case of low cholesterol content (0.6%), the rods do not tend to aggregate as much but the cholesterol concentration on their surface is not sufficient to facilitate a strong attachment to the GUVs membranes.

**Figure 5:**
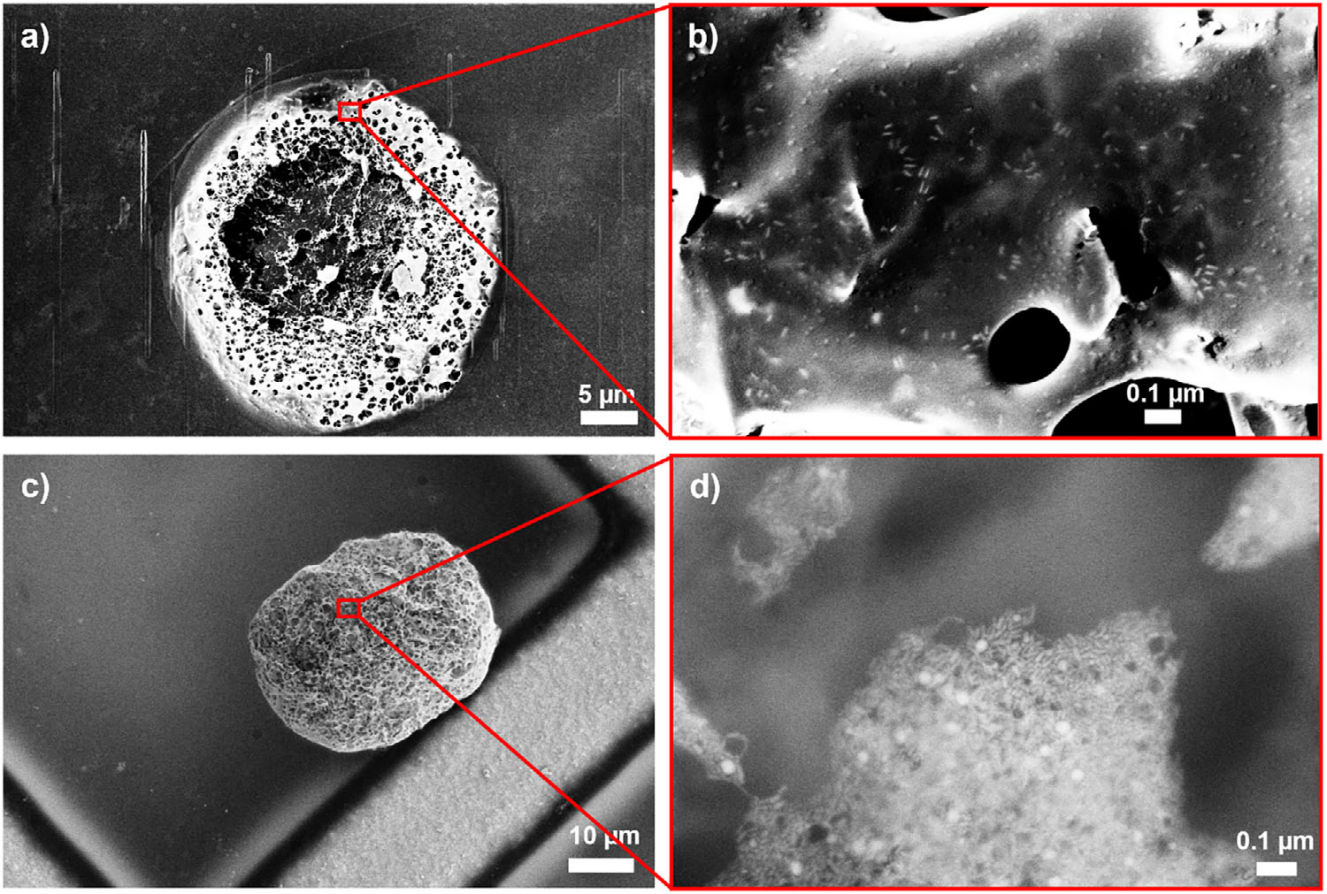
CryoSEM micrographs of GNR‐functionalized GUVs showing individual versus aggregate binding depending on the cholesterol content of the GNRs. Panels (a) and (b) show a GUV sample with GNR 1 (16.1% Chol), panels (c) and (d) show a GUV sample with GNR 3 (3.2% Chol). Panels (a) and (c) provide a wider view of the GUVs, whereas (b) and (d) show a magnified view of the membrane surface and GNR distribution on the GUV membrane.

To confirm this hypothesis, we conducted cryo‐SEM measurements to study the details of the nature of GNR‐GUV interactions. **Figure** **5**a,c shows representative cryoSEM micrographs of GUVs functionalized with GNRs containing 16.1 or 3.2% cholesterol, respectively. The corresponding magnified micrographs (Figure 5b,d) show distinct attachment behaviors. GNR 1 (16.1% Chol) attached primarily as individual nanorods, showcasing the few GNRs that remained in solution and were not washed away during purification. GNR 3 (3.2% Chol), on the other hand, attached to the GUV membrane in aggregates. Although cryo‐SEM captures static snapshots, Chol‐PEG‐SH‐tethered GNR clusters on fluid GUV membranes are expected to be laterally mobile. Previous reports on the diffusion coefficient (D) of cholesterol‐anchored nano‐objects on lipid bilayers indicate values of ≈0.5–1 µm^2^ s^−1^ for small, singly anchored constructs (e.g., a DNA origami brick of 22 × 65 nm). In our study, clustering and higher anchor multiplicity are expected to reduce D into the sub‐µm^2^ s^−1^ range.^[^ ^106^ ^]^ Functionally, these membrane clusters define loci of localized photothermal transduction under NIR; release is therefore spatially confined and transient, consistent with short‐lived packing defects at GNR‐membrane contacts. In isotonic media, solute efflux is driven by luminal‐external concentration differences and is osmotically balanced by counter‐fluxes, preserving overall vesicle integrity. In sum, these results support our hypothesis about the cholesterol‐dependent interplay, wherein excessive cholesterol drives formation of large aggregates (limiting membrane availability) and insufficient cholesterol restricts GNR attachment to the GUV membrane.

Although our optimum (3.2 mol% cholesterol) was obtained empirically, a simple working picture helps rationalize why a low, single‐digit percentage is a plausible concentration. First, each cholesterol‐terminated ligand acts as a hydrophobic anchor that partitions favorably into lipid bilayers; single‐molecule force spectroscopy and simulations report substantial extraction barriers and nonpolar stabilization of cholesterol in membranes.^[^ ^107^ ^]^ Second, as the fraction of cholesterol ligands on a GNR increases, the expected number of anchors that insert simultaneously within the contact area rises; multivalent frameworks show that binding can then strengthen nonlinearly and even display sharp thresholds with ligand/receptor availability.^[^ ^108^ ^]^ Third, at higher densities, anchors begin to compete and crowd, and cholesterol tags are known experimentally to promote aggregation or reduce effective membrane engagement depending on number, spacing, and spacer design—effects that can limit further gains.^[^ ^109^ ^]^ Together, these opposing trends—favorable insertion and multivalent gain at low‐moderate densities versus crowding/aggregation penalties at higher densities—are consistent with a broad optimum at a few mol%. This view yields testable predictions: the optimum should shift with spacer length/flexibility, membrane order/composition, and contact area (particle size/geometry), offering a route to tune adhesion without exhaustive empirical screening.

### 2.6 Triggered Release of Ampicillin for Antibacterial Applications

Following the detailed characterization of GNR‐mediated release, we set out to demonstrate the practical application of the GNR‐functionalized GUVs. Toward this end, ampicillin‐loaded GUVs were produced in a similar manner as the GUVs loaded with calcein, SUVs, or beads. *E. coli* was chosen as a model organism due to its well‐characterized sensitivity to ampicillin. To set out the experimental conditions, such as initial ampicillin concentration, we first investigated the minimal concentration essential for bacterial inhibition at an OD_600_ of 0.15 (Figure S11, Supporting Information). The results revealed that final ampicillin concentrations above 100 µg mL^−1^ suffice. Moreover, we based the determination of the ampicillin concentration on an encapsulation efficiency of ≈3%, a release efficiency of ≈67% for GNR 3 (3.2% Chol), and spontaneous leakage of ≈20% from nonilluminated GNR 3 (3.2% Chol) samples. As a result, we set the initial ampicillin concentration to 90 mg mL^−1^ (see the Experimental Section; and the Supporting Information).

To test the efficiency of NIR‐triggered ampicillin release, we incubated *E. coli* with ampicillin‐loaded GNR‐functionalized GUVs and illuminated the mixture with NIR light. Following the illumination, we plated the entire suspension on agar to assess bacterial growth. The results revealed that 2 min of illumination were sufficient for bacterial inhibition (Figure S12c, Supporting Information). In control experiments with the ampicillin‐loaded GUVs that were not illuminated with NIR light, only partial bacterial inhibition was observed due to spontaneous leakage (≈20%) (Figure S12, Supporting Information). Controls with ampicillin‐loaded GUVs lacking GNRs showed no difference between illuminated and nonilluminated conditions, indicating that drug release required GNR‐mediated heating. However, the level of bacterial inhibition in these controls was unexpectedly high, raising concerns about possible unspecific effects, such as ampicillin release during sample incubation at 37 °C or interactions between GUVs and the agar substrate (e.g., osmotic stress or local rupture).^[^ ^110^ ^]^ Although this setup closely resembled an *in vivo*‐like application, overlapping side effects—such as potential osmolarity mismatch, gel–air desiccation, direct photothermal effects on bacteria, and diffusive carryover of free drug—make it difficult to attribute bacterial inhibition solely to NIR‐triggered ampicillin release.

One possible improvement for the experimental design could involve the separation of bacteria from intact GUVs after illumination. However, due to a similar sedimentation behavior of bacteria and ampicillin‐loaded GUVs under centrifugation, a clean separation is technically challenging and prone to cross‐contamination. To overcome these issues, we implemented an adjusted sequential protocol in which ampicillin‐loaded, GNR‐functionalized GUVs were first illuminated to trigger release and centrifuged to remove intact vesicles. The resulting supernatant—containing only the released ampicillin—was then incubated with bacteria prior to plating. To avoid excessive thermal exposure, we focused on shorter illumination times and additionally explored 1 and 3‐min intervals to assess the time‐dependence of the release behavior (Figure S10, Supporting Information).

**Figure 6:**
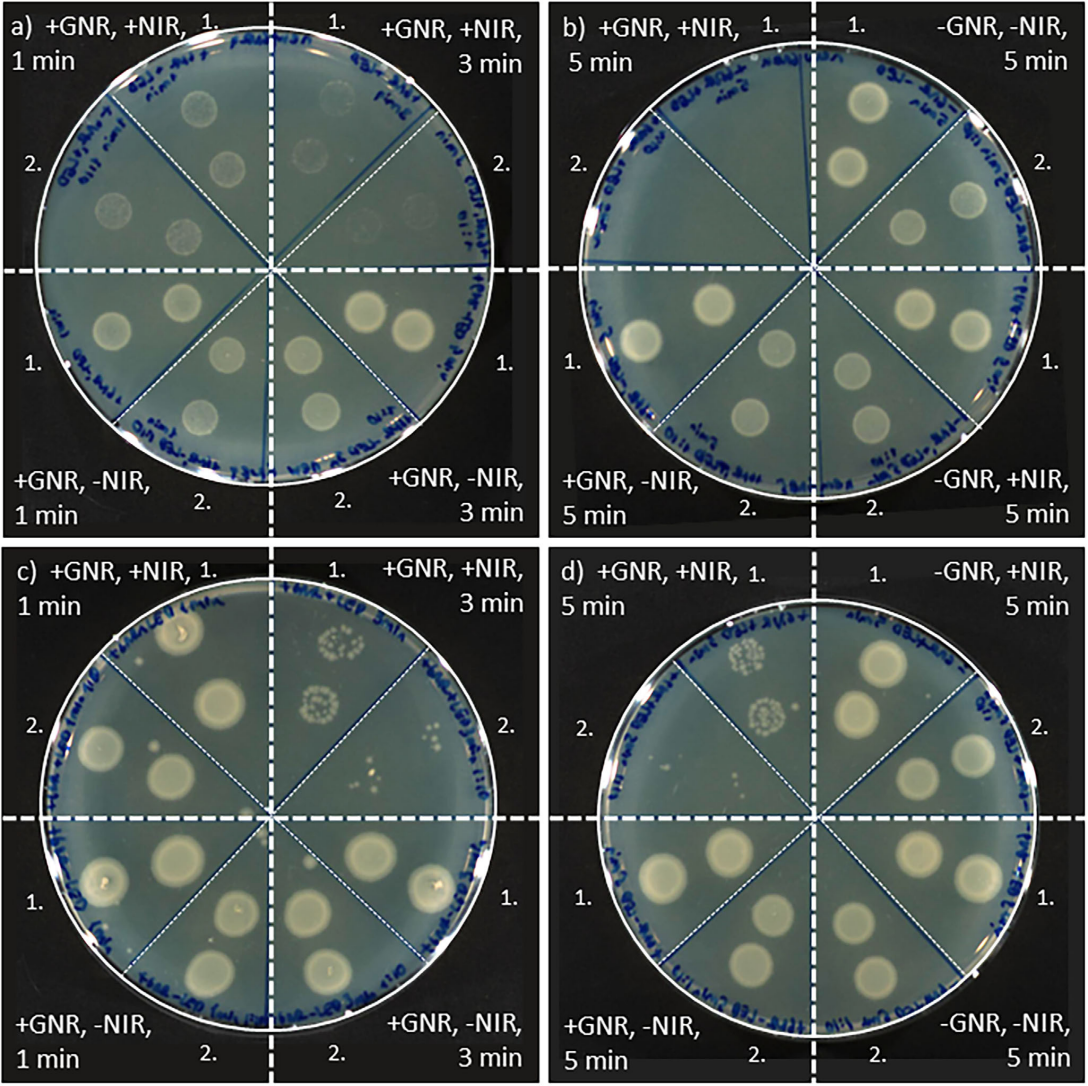
Bacterial viability following NIR‐triggered ampicillin release and GNR‐mediated heating. a,b) *E. coli* (laboratory strain) plated on LB agar after treatment with supernatants from ampicillin‐loaded, GNR‐functionalized GUVs following NIR light illumination for 1, 3, or 5 min, compared with nonilluminated (dark) controls. Supernatants from ampicillin‐loaded GUVs lacking GNRs were included with and without NIR light illumination. c,d) To assess heating‐only effects, *E. coli* bacteria were exposed to empty GNR‐functionalized GUVs under the same illumination times, alongside the corresponding dark controls; samples without GNRs served as additional controls. Numbers next to plates indicate dilutions of the treated suspension in LB prior to plating: 1 = undiluted, 2 = 1:10. Illumination (all panels): CW NIR LED, 4 W cm^−^^2^; durations are as indicated in Figure 6 next to the sample. GNR 3 (3.2% Chol) was used for all GNR‐functionalized GUV samples.

**Figure** **6**a,b shows a time‐dependent antibiotic response: after 1 min of illumination bacterial growth was still visible; after 3 min it was reduced; and after 5 min it was completely suppressed (Figure 6a,b). In contrast, nonilluminated GNR‐GUV samples showed no significant inhibition, indicating that passive leakage (≈20%) was insufficient to kill the bacteria. Notably, the antibacterial activity remained effective even after 5 min of illumination, suggesting that the elevated temperature stemming from GNR heating did not impair the efficacy of the ampicillin. An additional benefit of this setup was the ability to isolate the thermal effect of the GNRs. To this end, bacteria were incubated with empty GUVs (without ampicillin), illuminated, and plated on agar. The results showed that heating alone—via GNR‐mediated photothermal effects—was sufficient to reduce bacterial growth after 3 or 5 min of illumination (Figure 6c,d), whereas 1 min of illumination had no noticeable effect. Control samples without GNRs did not display bacterial death, confirming that the heat effect depended on both the presence of GNR and sufficient illumination time. Together, these findings demonstrate a dual antimicrobial mechanism: antibiotic release and localized photothermal heating. This highlights the potential of the GNR‐GUV system as an effective, controllable therapeutic platform.

## 3 Conclusion

In this work, we developed a photothermally‐responsive cargo delivery platform based on GNR‐functionalized GUVs for controlled NIR‐triggered release. GUVs encapsulating various cargo were generated via the emulsion transfer method. Anchoring of GNRs to the GUV membrane was achieved through the functionalization of GNRs with cholesterol. The optimization of the cholesterol content on the GNRs was an essential step: insufficient cholesterol led to poor GNR membrane attachment, whereas excessive amounts promoted GNR aggregation in solution, thus reducing their membrane availability. Optimal GNR functionalization at a concentration of 3.2% cholesterol achieved the highest release efficiency, peaking at ≈70% after 10 min of NIR illumination. GNRs with higher and lower concentrations of cholesterol, just like shorter illumination times, resulted in significantly lower release efficiency. The system's functional validation was demonstrated by illuminating ampicillin‐loaded GUVs with NIR light in the presence of *E. coli*. Bacterial killing was observed already after 3 min of illumination, which can be attributed to the release of encapsulated ampicillin. In contrast, nonilluminated control samples showed no antibacterial activity. Notably, GNR‐mediated photothermal heating alone—without the release of an antibiotic—was also capable of reducing bacterial viability at extended illumination times, indicating a dual mechanism of action. From a translational perspective, key challenges include immune recognition and macrophage uptake of GNR‐functionalized vesicles, rapid systemic clearance with hepatic/splenic sequestration, and the long‐term fate of gold nanoparticles. These risks could be mitigated through stealth strategies or cell‐derived membranes (e.g., ghost red blood cells (GRBC)‐mimetic vesicles), as well as by implementing the trigger in high‐loading LUV formats. A full evaluation of pharmacokinetics, pharmacodynamics, and safety lies beyond the present scope and will be addressed in future studies.

## 4 Experimental Section

### Reagents and Solvents

The lipids, namely DOPG, DOPC, and 1,2‐dioleoyl‐*sn*‐glycero‐3‐phosphoethanol‐amine‐*N* liss‐amine rhodamine B sulfonyl ammonium salt (LissRhod PE), were purchased from Avanti Polar Lipids, Inc. 5 kDa FITC‐PEG‐SH was ordered from Creative PEGWorks, 2 kDa Cholesterol‐PEG‐SH from Abbexa, and TRIS‐HCl from Carl Roth. N‐Decane, 10x Phosphate Buffered Saline (PBS), and FluoSpheres Carboxylate‐Modified yellow‐green fluorescent beads (0.2 µm) were ordered from Fisher Scientific. Triton X‐100 was ordered from AppliChem. Cell Viability Assay Kit (fluorometric blue) was ordered from Abcam. Milli‐Q water was used for the experiments, unless otherwise specified. The rest of the reagents were purchased from Sigma‐Aldrich and used without further purification.

### Confocal Laser Scanning Microscopy

Microscopy experiments were performed using a Zeiss LSM 880 microscope (Carl Zeiss AG, Germany) with a 63x oil objective (Plan‐Apochromat 63x/1.4 Oil DICII, Carl Zeiss AG, Germany) and a 20x objective (Plan‐Apochromat 20×/0.8 DICII Carl Zeiss AG, Germany). Microscopy measurements of GUVs were performed at room temperature. Recorded images were adjusted for brightness and contrast and analyzed using Zen 3.4 (Blue edition) software.

### Cryo‐Scanning Electron Microscopy (Cryo‐FE‐SEM)

Cryo‐FE‐SEM was conducted with ZEISS Gemini SEM 560 to characterize GNR‐GUVs under cryogenic conditions (‐150 °C). Highly concentrated samples were applied to TEM grids and left for 5 min to allow sedimentation. Excess dispersant was removed, and the grids were shock‐frozen in liquid nitrogen. Sublimation of liquid residues was performed for 45 min at ‐90 °C using the EM Sample Preparation Freeze Fracture System Leica EM ACE900 from Leica Microsystems GmbH, Wetzlar, Germany. To prevent charging, samples were coated with a 5 nm carbon layer before imaging. Observations were conducted at a working distance of 5–6 mm using in‐lens and SE2 detectors with acceleration voltages of 2–10 kV.

### Bending Rigidity

For bending rigidity experiments, GUVs were prepared via electroformation^[^ ^41^ ^]^ to avoid potential contribution of oil pockets influencing the fluctuation spectra. A 10 µL lipid solution in chloroform was spread onto the conductive surfaces of two indium‐tin‐oxide (ITO)‐coated glass plates, dried under vacuum for 1 h, and assembled in a swelling chamber with a 2 mm‐thick Teflon spacer. The lipid layer was rehydrated with 20 mm sucrose, and a 10 Hz, 1.6 Vpp AC electric field was applied for 1 h to induce GUV formation. GUVs were harvested by pipetting and diluted 1:10 in 22 mm glucose for fluctuation analysis. These low osmolarities prevent gravity‐induced distortions in the fluctuation spectra, while simultaneously stabilizing the vesicles osmotically, maintaining a constant volume, and promoting their sedimentation to the chamber bottom for easier observation. GNRs were added directly to GUVs (5 µL of 1:1 diluted GNR to 100 µL GUVs) ensuring that the solutions are isotonic. The osmolarity of all solutions was carefully matched using an Osmomat 3000 freezing point osmometer (Gonotec, Berlin, Germany). Control GUVs were imaged as prepared. Imaging was performed using a Zeiss AxioObserver.D1 microscope with a 63×1.2NA water immersion objective and a pco.edge sCMOS camera. Phase contrast image sequences of 3000 frames were recorded for 13 GUVs from each sample at 25 frames per second with an exposure time of 200 µs. Vesicle contour detection and fluctuation analysis was conducted using a software developed by Gracia et al.,^[^ ^88^ ^]^ taking various constraints and limitations into account.^[^ ^111^ ^]^

### Preparation of Gold Nanorods

Gold nanorods were prepared using a silver‐assisted seed‐mediated growth method.^[^ ^61^ ^]^ The protocol was adjusted to prepare the GNRs with an absorbance maximum of around 750 nm. The seed solution was prepared HAuCl_4_ (0.01 m, 0.25 mL) to a solution of CTAB (0.1 m, 9.75 mL) in a water bath at ≈25–27 °C with gentle stirring. Then, a freshly prepared ice‐cold solution of NaBH_4_ (0.01 m, 0.6 mL) was quickly added to the mixture and stirred with a magnetic stirrer for 3 min until the solution changed its color to dark brown. The solution was incubated for 2 h to allow the growth of gold seeds. To prepare the growth solution, the CTAB solution (0.1 m, 90 mL) was kept in a water bath at ≈25–27 °C. HAuCl_4_ (0.01 m, 5 mL), AgNO_3_ (0.01 m, 0.65 mL), and HCl (1 m, 2 mL) were sequentially added to the CTAB. After thorough mixing, ascorbic acid (0.1 m, 0.8 mL) was added to the solution and the mixture stirred until it turned colorless. Finally, 10 mL of the seed solution was added to the growth solution, briefly mixed (30 s,) and left in the water bath overnight without stirring. Afterward, the GNR solution was centrifuged for 15 min at 16060 x g (Heraeus, rotor radius 8.5 cm), excess CTAB was removed, and the sample resuspended with water to a final volume of 10 mL. The GNRs were refrigerated at 4 °C. The absorbance and Zeta potential of the GNRs were checked with a Tecan Spark plate reader and a Malvern Zetasizer. Data processing of the absorbance curves was done in Origin utilizing the FFT function to remove high frequency noise. The size and aspect ratio of the nanorods was determined via TEM or SEM imaging.

The final GNR concentration was estimated according to Scarabelli's method.^[^ ^112^ ^]^ The GNR concentration was determined based on the absorbance value at 400 nm. SEM micrographs of the GNRs provided estimated dimensions of ≈28 nm in length and 10 nm in width. For calculation purposes, the nanorods were approximated as cylindrical in shape. Using this estimated volume and the known density of gold (ρ_gold_ = 1.932 × 10⁴ kg m^−^^3^), the mass of a single GNR was calculated as m_GNR_ = 6.070 × 10^−20^ kg, corresponding to *n* = 3.081 × 10^−19^ mol per GNR. The molar extinction coefficient of gold was used at 400 nm (*ε*_400_ = 2.02 × 10^3^ m ^−1^ cm^−1^). The final concentration of CTAB‐GNRs was found to be around 4.7 × 10^12^ particles mL^−1^.^[^ ^113^ ^]^

### Coating of Gold Nanorods with mPEG(2000)‐SH, Chol‐PEG(2000)‐SH, and FITC‐PEG(5000)‐SH

The procedure of coating nanorods was adapted and optimized from a protocol by Zhang et al.^[^ ^67^ ^]^ (Figure S2, Supporting Information). An aliquot of previously synthesized gold nanorods (0.2 mL) was slightly heated (25–27 °C) to dissolve the CTAB, centrifuged for 15 min at 13 000 rpm to remove excess CTAB, and the pellet afterward redispersed in water. For PEG‐coated GNRs: mPEG‐SH was added at a final concentration of 2.5 mm along with 400 µL TRIS buffer (50 mm, pH 3). 50 mm TRIS buffer (pH 3) was made by adding 0.788 g TRIS‐HCl and 1 mL of 10% SDS into 100 mL of water. The pH was adjusted to 3.0 with 1 m HCl. The buffer was then kept at room temperature. The GNR sample was shaken at room temperature for 30 min at 300 rpm. Finally, the sample was centrifuged twice for 15 min at 16 000 g to remove excess components and resuspended in water and stored at 4 °C. For FITC‐functionalized samples: mPEG(2000)‐SH (3 mm), FITC‐PEG(5000)‐SH (2 mm), and Chol‐PEG(2000)‐SH (5 mm) were added at ratios of 20:1:2.5 to achieve a final concentration of 2.5 mm. A slightly higher total PEG concentration was used to compensate for potential steric hindrance and competition between FITC‐PEG(5000)‐SH and Chol‐PEG(2000)‐SH molecules during functionalization. A control sample was prepared without Chol‐PEG(2000)‐SH while keeping the same final concentration of components.

For GNR samples 1–4: The mPEG(2000)‐SH to Chol‐PEG(2000)‐SH ratio is given in Table S1 (Supporting Information). An aliquot of concentrated CTAB‐GNRs (1 mL) was centrifuged to remove excess CTAB, after which mPEG(2000)‐SH (3 mm) and Chol‐PEG(2000)‐SH (5 mm) were added to reach a final concentration of 1.65 mm. The total volume of all components was adjusted to 500 µL using 50 mm TRIS buffer (pH 3).

### Preparation of Lipid Vesicles (GUVs, SUVs)

GUVs were produced using a water‐in‐oil emulsion transfer method.^[^ ^114^ ^]^ The lipid‐oil suspension was made by drying lipid films of 80% DOPC and 20% DOPG dissolved in chloroform (final lipid concentration: 643 µm per 1 mL silicon oil). After drying, 60 µL of n‐Decane was added to the lipids and mixed under N_2_. Then, 940 µL of silicon oil was added and the mixture was briefly vortexed for 10 s. The Outer Buffer was prepared by mixing 50 µL of 50 mm imidazole (pH 7.64, adjusted with HCl), 128 µL of 1 m sucrose, and 322 µL of milliQ water, resulting in a final osmolarity of ≈300 mOsm. Subsequently, 400 µL of the lipid‐oil mixture was gently added to the Outer Buffer without touching the tube walls, leaving the remaining mixture under N_2_. The solution was incubated for 45–90 min to allow lipid monolayer formation. The Inner Buffer (10 µL) for single‐compartment GUVs consisted of 3.3 µL OptiPrep, 1 µL 10x PBS, and 5.7 µL water. It was added to the lipid‐oil mixture using a glass pipette and mixed thoroughly (15–20 times). The resulting mixture was carefully layered onto the Outer Buffer as a lipid monolayer and centrifuged at 380 g for 3 min. After centrifugation, the top oil phase was removed and the GUVs were briefly vortexed for resuspension. To purify the sample from residual oil, the aqueous phase of the vortexed emulsion was carefully transferred to a fresh Eppendorf tube, followed by centrifugation and supernatant exchange. The final volume per GUV sample was adjusted to 500 µL and stored at 4 °C for use within 1 day. For experiments involving multiple conditions (e.g., release assays), samples were pooled and evenly divided into 250 µL aliquots.

### Preparation of Cargo‐Containing and Multicompartment GUVs

Cargo‐containing GUVs were also produced as described above with adjustments to component volumes to maintain osmolarity (≈300 mOsm; in the case of ampicillin‐loaded GUVs 430 mOsm). The Inner Buffer included either 5 mm calcein, 90 mg mL ampicillin, or 1 µL of fluorescent beads (FluoSpheres carboxylate, 0.2 µm, yellow‐green). For multicompartment GUVs, the Inner Buffer was modified to include 1 µL of SUVs (Small Unilamellar Vesicles) along with 3.3 µL of OptiPrep, 1 µL of 10x PBS, and 4.7 µL of water.

The encapsulation efficiency of calcein‐loaded GUVs was estimated based on the obtained calcein concentration from the calibration curve (Figure S8a, Supporting Information) and the following formula:

(1) $$EE\;\left(\%\right)=\frac{C_{\mathrm{calcein}}*V_{\mathrm{Outer}\;\mathrm{Buffer}}}{C_{\mathrm{calcein}\;\mathrm{in}\;\mathrm{Inner}\;\mathrm{Buffer}}*V_{\mathrm{Inner}\;\mathrm{Buffer}}}*100\%$$

### Preparation of SUVs

SUVs were prepared by mixing lipids in chloroform to achieve the desired molar ratios, followed by drying under vacuum for at least 30 min. The lipid films were resuspended in 1x PBS at the desired concentration and left to swell for 30 min at room temperature. Afterward, the solution was shaken for at least 5 min to facilitate liposome formation. Homogeneous SUVs were obtained by passing the suspension through a 100 nm polycarbonate filter (Avanti Polar Lipids, Inc.) 21 times using an extruder. The SUVs were stored at 4 °C until further use.

### Attachment of Gold Nanorods to GUVs

GNRs were diluted 1:1 with water, and 5 µL of the suspension was added to 100 µL of GUVs, followed by gentle mixing. To remove unbound GNRs, the sample was centrifuged for 3 min (380 g) and resuspended in the GUV Outer Buffer. For imaging experiments, the sample was placed in a silicon imaging chamber coated with 1% BSA and imaged using a confocal microscope. For calcein release experiments, the prepared solution was transferred to a 1.5 mL Eppendorf tube and illuminated with the LED setup.

### Release of the Cargo from the GNR‐Functionalized GUVs

GUVs containing either calcein or fluorescent beads were prepared as described above, pooled, and divided into 250 µL aliquots in 1.5 mL Eppendorf tubes to ensure consistent GUV concentrations across samples. GNRs were added to the GUVs and purified, with control samples processed in parallel for equal treatment. To minimize leakage and time‐dependent variability, GNRs were added just before NIR illumination. GUVs were illuminated for 10 min unless stated otherwise. After illumination, samples were centrifuged and fluorescence measurements were performed using the supernatant and the pellets resuspended in the Outer Buffer with 1% Triton X. Control samples without GNRs were also centrifuged to ensure equal treatment. Additional controls without NIR illumination as well as samples with and without GNRs were included to separately evaluate the effects of NIR light and GNRs. The pellet was resuspended in 250 µL of Outer Buffer with 1% Triton X to lyse remaining GUVs. Fluorescence values were converted to cargo concentrations using calibration curves (Figure S8a,b, Supporting Information), and the percentage of release was calculated using this formula:

(2) $$\begin{array}{c} \mathrm{Release}\;\%=\frac{\mathrm{Calcein}\;\mathrm{concentration}\;\mathrm{in}\;\mathrm{supernatant}}{\mathrm{Total}\;\mathrm{Calcein}\;\mathrm{concentration}\;\left(\mathrm{supernatant}+\;\mathrm{pellet}\right)\;}\;*100\% \end{array}$$

The error was calculated as standard deviation of the release % of individual samples.

### Temperature Measurements

Temperature measurements were carried out to monitor heat generation during NIR illumination. Samples (250 µL) were placed in open 1.5 mL Eppendorf tubes and illuminated under standard conditions. A GMH 3210 digital thermometer (Greisinger Scientific) equipped with a thermocouple probe was used to record the temperature at defined time intervals. For GNR‐functionalized GUV samples measurements were taken every minute. For GNR‐only samples (in water) the temperature was recorded every 10 s for the first 2 min, followed by 1‐min intervals. All samples were prepared according to the standard release protocol, with 5 µL of GNR solution added to 100 µL of either GUV suspension or water.

### Release of Ampicillin and Killing of Bacteria

*Escherichia coli DSM 613* was routinely cultured on 1.5% w/v Standard I agar plates (15 g L^−1^ peptone, 3 g L^−1^ yeast extract, 6 g L^−1^ NaCl, 1 g L^−1^ glucose, 12 g L^−1^ agar, pH 7.5 ± 0.2) from glycerol stocks. For liquid culture bacteria were grown in yeast‐based nutrient broth (10 g L^−1^ tryptone, 5 g L^−1^ yeast extract, 10 g L^−1^ NaCl, pH 7.0 ± 0.2) with aeration at 37 °C. Exponentially growing cells were harvested, resuspended in fresh nutrient broth, and diluted to an OD_600_ of 0.3 (≈2.4 × 10⁸ cells mL^−1^).

Ampicillin‐loaded GUVs were prepared via the emulsion transfer method as described in the GUV preparation section. Due to visibly reduced yield, two GUV batches were combined per condition and concentrated into 200 µL of the outer phase. All GUV batches were pooled to ensure a consistent concentration across samples. To estimate the concentration of encapsulated ampicillin in the final GUV sample, the following calculation was performed: 90 mg mL^−1^ of ampicillin was added to 10 µL of Inner Buffer per GUV production. Since two GUV batches were combined, the total volume of the Inner Buffer was 20 µL, resulting in a total input of 1.8 mg of ampicillin. The GUVs were subsequently resuspended in 200 µL buffer. Assuming an encapsulation efficiency of ≈3%—based on calcein release data and the similar small molecular weights of ampicillin (349.41 g mol^−1^) and calcein (622.55 g mol^−1^)—, the amount of encapsulated ampicillin was estimated to equal 54 µg. When distributed in the final sample volume of 200 µL, this corresponds to a concentration of −270 µg mL^−1^ of encapsulated ampicillin. Thus, considering a release efficiency of ≈67%, the expected amount of released ampicillin would be ≈180 µg mL^−1^. Additionally, accounting for the observed spontaneous leakage of ≈20%, the amount passively released without illumination would be ≈54 µg mL^−1^.

### Initial Experimental Setup (Coincubation with GUVs and Bacteria)

An aliquot of GUVs (200 µL) was incubated with 15 µL of GNRs, followed by purification via centrifugation. The pellet was resuspended in 100 µL of LB medium (osmolarity adjusted to ≈430 mOsm using 1% v/v 10× PBS). Control samples without GNRs were processed identically. Subsequently, 100 µL of *E. coli* suspension (OD_600_ = 0.3) was added to each sample. The mixtures were illuminated with NIR light for 1 or 2 min, while nonilluminated controls were kept at room temperature. All samples were then incubated at 37 °C for 20 min with gentle shaking at 300 rpm. Eppendorf tubes were sealed with parafilm to ensure sufficient aeration. Following incubation, 10 µL of each sample (undiluted and diluted 1:10 with final cell concentrations of 1.2×10⁸ and 1.2×10⁷ cells mL^−1^, respectively) was spotted onto Standard I agar plates. Plates were dried at 37 °C for 30 min, incubated overnight at room temperature, and then further incubated at 37 °C for 2 h prior to imaging.

### Improved Experimental Setup (GUV Removal Prior to Bacterial Exposure)

An aliquot of GUVs (200 µL) was incubated with 15 µL of GNRs, followed by purification via centrifugation. The pellet was resuspended in 100 µL of LB medium (≈430 mOsm). Samples were then illuminated with NIR light for 1, 3, or 5 min; nonilluminated controls were incubated at room temperature. Samples were subsequently centrifuged at 2000 rpm for 3 min to remove intact GUVs. The resulting supernatant (100 µL), containing only the released ampicillin, was added to 100 µL of *E. coli* suspension (OD_600_ = 0.3) and incubated at 37 °C for 20 min with gentle agitation at 300 rpm. Tubes were sealed with parafilm for aeration. After incubation, 10 µL of each sample (undiluted and 1:10 dilution) were spotted on agar plates as described above, dried at 37 °C for 30 min, and incubated overnight at room temperature, followed by 6 h at 37 °C before imaging.

Control samples without ampicillin were prepared using the initial experimental design to specifically investigate the effect of GNR‐mediated photothermal heating on bacterial viability. In these samples *E. coli* was coincubated with GUVs (without ampicillin), followed by NIR illumination and plating as described in the initial setup.

### Statistical Analysis

*Preprocessing*. Absorbance spectra of GNRs were smoothed in Origin (FFT, 10 pts.) to suppress high‐frequency noise; no further transformations were applied. No outliers were excluded.

*Data presentation*. Unless stated otherwise, data are reported as mean ± SD. Figure captions specify what the center and error bars represent.

*Sample size*. For zeta potential, cargo‐release efficiency, GUV size distributions, cell‐viability, and temperature measurements, *N* = 3−5 independent samples per condition (exact *N* is given in each figure caption). For size distributions, *N* denotes the number of individual GUVs analyzed (reported per panel).

*Statistical tests*. Cargo‐release efficiency was compared per GNR formulation between NIR‐illuminated and nonilluminated groups using two‐sided, unpaired Welch's *t*‐tests. Significance is indicated with stars in the figures as: ns (*p* ≥ 0.05), * (*p* < 0.05), ** (*p* < 0.01), *** (*p* < 0.001). Multiple pairwise comparisons were performed within some panels; *p* values are unadjusted. Exact *p* values are provided in Table S2 (Supporting Information).

*Software*. OriginPro (OriginLab) was used for curve processing and statistics; Excel (T.TEST) was used for confirmation of *t*‐tests. GUV sizing was performed in Fiji (ImageJ) by manual ROI selection of approximately spherical vesicles; equivalent circular diameters were computed from ROI areas.

## Conflict of Interest

The authors declare no conflict of interest.

## Supporting information



Supporting Information

## Data Availability

The data that support the findings of this study are available in the supplementary material of this article.
