# Supplementary material for: Design of a Trigger‐Responsive Photothermal Vesicle‐Based Cargo Delivery Platform
Source: Small. 2025 Nov 12;21(51):e05852. doi: 10.1002/smll.202505852 (PMC12723353; doi:10.1002/smll.202505852)
Supplement: Supplementary file 1 — Supporting Information [file SMLL-21-e05852-s001.docx]

**Supplementary Information**

**Design of a Trigger-Responsive Photothermal Vesicle-Based Cargo Delivery Platform**

*Anastassiya Schramm^†,§^, Nina F. Conzelmann^†^, Ann-Kathrin Gelmroth^†^, Julia Köberle^†^, Stefan Schramm^||^, Rumiana Dimova^#^, Ilia Platzman*^,†^, and Joachim Spatz*^,†,‡,§,^***^┴^**

^†^Department of Cellular Biophysics, Max Planck Institute for Medical Research, Jahnstraße 29, 69120 Heidelberg, Germany

^‡^Institute for Molecular Systems Engineering and Advanced Materials, Heidelberg University, Im Neuenheimer Feld 225, 69120 Heidelberg, Germany

^§^Max Planck School Matter to Life, Jahnstraße 29, 69120 Heidelberg, Germany

^||^University of Applied Sciences Dresden, Friedrich-List-Platz 1, 01069 Dresden, Germany

^#^Max Planck Institute of Colloids and Interfaces, Science Park Golm, Potsdam 14476, Germany

**^┴^** Max Planck-Bristol Center for Minimal Biology, University of Bristol, Bristol BS8 1TD, U.K

Corresponding authors: ilia.platzman@mr.mpg.de, spatz@mr.mpg.de

**Preparation and Surface Modification of Gold Nanorods**


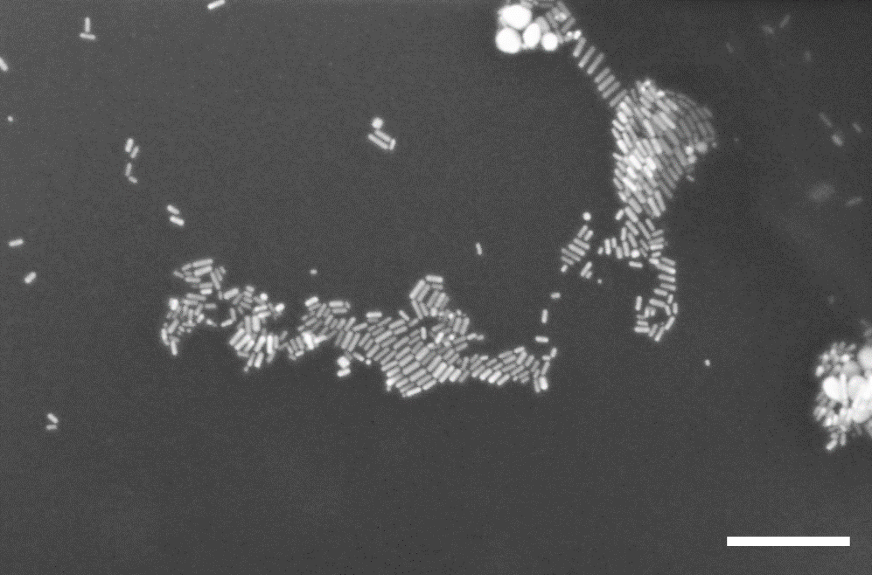


**Figure S1.** SEM micrograph of the CTAB-GNR. The scale bar is 200 nm.

**Table S1.** New types of GNRs coated with decreasing concentrations of cholesterol-PEG-SH.

| **GNR type** | **Ratio of mPEG(2000)-SH** | **Ratio of Chol-PEG(2000)-SH** | **% Chol-PEG(2000)-SH** |
| --- | --- | --- | --- |
| GNR 1 | 13.0 | 2.5 | 16.1 |
| GNR 2 | 14.0 | 1.5 | 9.7 |
| GNR 3 | 15.0 | 0.5 | 3.2 |
| GNR 4 | 15.4 | 0.1 | 0.6 |


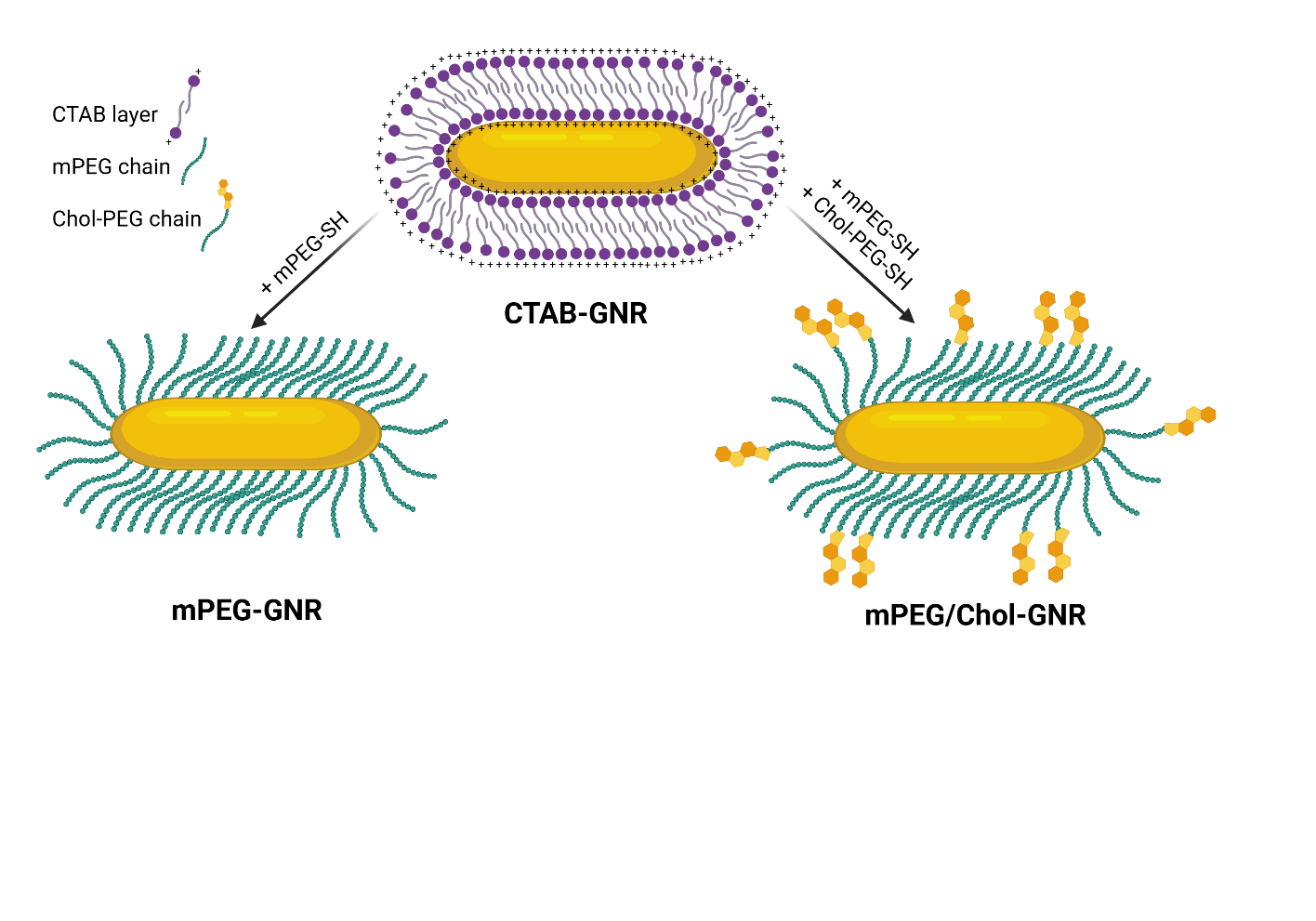


**Figure S2.** Schematic representation of the ligand exchange process: Replacing the initial CTAB surface coating with either mPEG-SH or a mixture of mPEG-SH and cholesterol-PEG-SH.

**Preparation of Single-Compartment and Multi-Compartment GUVs**


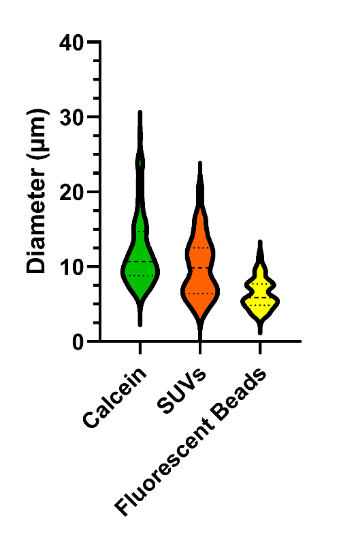


**Figure S3.** Statistical analysis of the size distribution of the GUVs encapsulating calcein, SUVs, or fluorescent beads. Number of GUVs used for the analysis: N=125 (calcein), N=56 (SUVs) and N=123 (fluorescent beads).

**Cell Viability Experiment**

The cell viability experiment was done with the help of the Cell Proliferation Assay Kit (Fluorometric Blue). The cell line used for the experiment was Ref52 wt. Cells were plated in triplicates in a 96-well plate at a concentration of 1.46*10^4^ cells/well (corresponds to 90 µl of cells/well). The following samples were compared: 1) pure cells, 2) cells with GUVs, 3) cells with GNR-GUVs, 4) cells with CTAB-GNRs, 5) cells with PEG-GNRs, and 6) cells with Tween 20. For the samples with “stimuli” (GUVs, GNRs or Tween 20), 10 µl of the “stimulant” was added to the cell plate and incubated overnight. Then, 10 µl (10% medium volume) of the dye from the kit was carefully added to the samples and incubated at 37°C, 5% CO_2_. After a 1 h incubation period, the fluorescence intensity was recorded at Ex. 530 nm and Em. 590 nm.

**
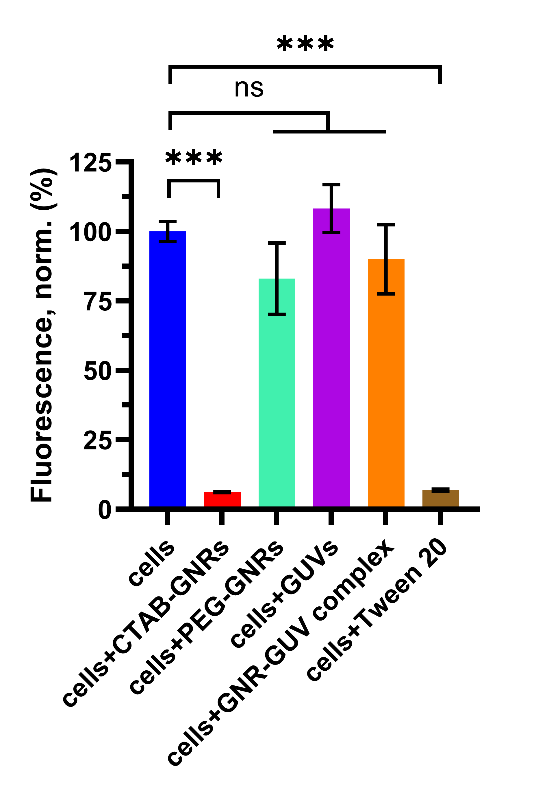
**

**Figure S4.** Cell viability test on *Ref52 wt* cells. Cells were incubated with CTAB-GNRs, mPEG GNRs, pure GUVs, and the GNR 2 (9.7% Chol)-GUV complex. Tween 20 was used as a negative control. After incubation the dye components from the cell viability kit were added and fluorescence was measured to quantify the number of living cells. N=3.

**Portable NIR LED Setup**

An LED-based system was chosen as a safer and more cost-effective alternative to traditional lasers, as it offers adjustable power levels and a broad range of experimental conditions. An advanced NIR LED system was engineered using high-power COB (Chip on Board) LEDs with a peak emission wavelength of 730 nm. The core of the LED module is a 100W COB LED, which is securely mounted onto an 80 mm aluminum cooling block. The attachment is facilitated by thermal conductive paste to ensure optimal heat transfer and stability and four M4 screws for firm securing. Active cooling of the system is achieved through the addition of a 12V, 80 mm fan, which is mounted onto the cooling block to provide continuous airflow, thus maintaining an efficient thermal management system (Figure S5).

For the optical setup, a 60° reflector was positioned in front of the COB LED to direct and enhance the emitted light, followed by the installation of a plano-convex PMMA lens that helps initially focus the light beam. This configuration ensures that the light is effectively concentrated before further collimation. A critical component of the system is a Thorlabs SM2F32-B collimator – modified by attaching an additional plano-convex PMMA lens to the collimator front – which is threaded onto the setup. This lens further focuses the light beam, particularly for applications requiring close-distance illumination. For experimental precision, the collimation ring is adjusted to a position where it just makes contact with the front PMMA lens, ensuring a consistent and optimal light focus. To accommodate the samples, adapters for 1.5 ml tubes or quartz cuvettes were designed that can easily be clipped onto the front of the system. This allows for straightforward sample handling and interchangeability during experiments. The system is equipped with two distinct power connectors: one dedicated to the operation of the 12V cooling fan, ensuring continuous thermal regulation, and the other for powering the high-power 100W COB LED. This dual power connector setup facilitates independent control of the cooling and illumination components, thereby enhancing the overall functionality and reliability of the LED system.


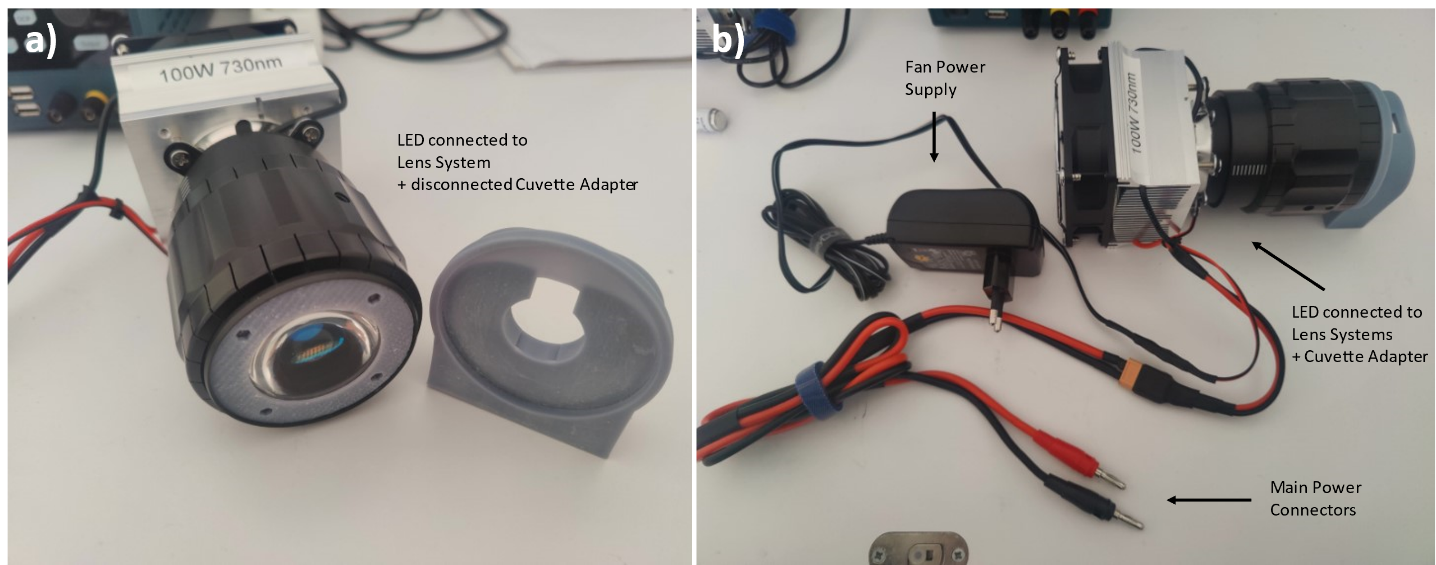


**Figure S5.** Portable NIR LED setup. (a) 100 W 730 nm COB LED setup connected to the modified Thorlabs SM2F32-B collimator next to the adaptor for cuvettes/Eppendorf tubes. (b) The entire system setup including the separate power connectors.

**Calibration Curves for the NIR LED**

The absolute light power output and power density of the LED light sources were measured using a Thorlabs S142C - Integrating Sphere Photodiode Power Sensor connected to a Thorlabs PM100A - Compact Power Meter Console. This detection is based on an integration sphere setup which is in accordance with the widely acknowledged standard way of calibrating the luminous flux of LEDs (CIE 127:2007, Chapter 6; DIN 5032-9). The setup used is rated by the manufacturer for a wavelength range between 350-1100 nm, optical power working ranges from 1 µW-5 W, and a peak optical power density of 2 kW/cm² with a measurement uncertainty of ±3% between 440-980 nm, according to the Thorlabs Protocol provided at:

https://www.thorlabs.com/_sd.cfm?fileName=18365-S01.pdf&partNumber=S142C. The entry port of the integration sphere has an area of 1.13 cm², which makes it suitable for measuring the luminous flux (i.e., optical light power) of the NIR LED setup.

To measure the light output of the high-power COB LED system, it was placed together with the optical system that is directly attached to the measurement setup. This most closely mimics the configuration later used in illumination experiments. A defined electrical power was applied to the LED to initiate its electroluminescence. After thermal equilibration the corresponding light flux was measured using the measurement setup described above. This procedure was repeated for several electrical power settings in order to record an electrical power / light power calibration curve. The used LED showed very good linearity in the light power range from 1 mW - 5000 mW. The power density was calculated by dividing the measured light power by the entry area of the integration spheres. By multiplying the power density by the total area of the LED, the absolute light output of the LED was calculated (Figure S6).


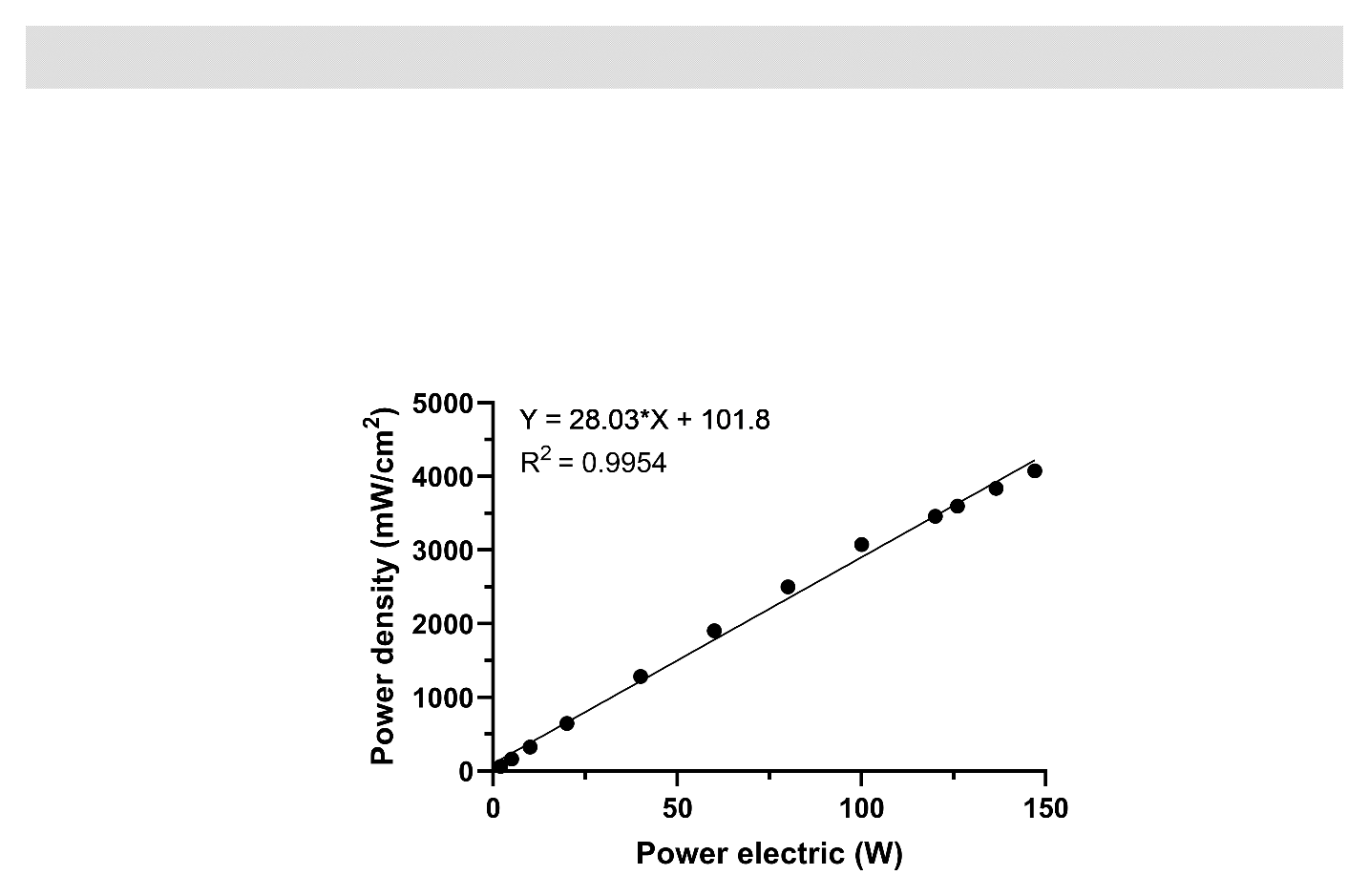


**Figure S6**. Calibration curve for calculating the light power density of the 730 nm LED.

**Release of the Cargo from the GNR-functionalized GUVs**

**
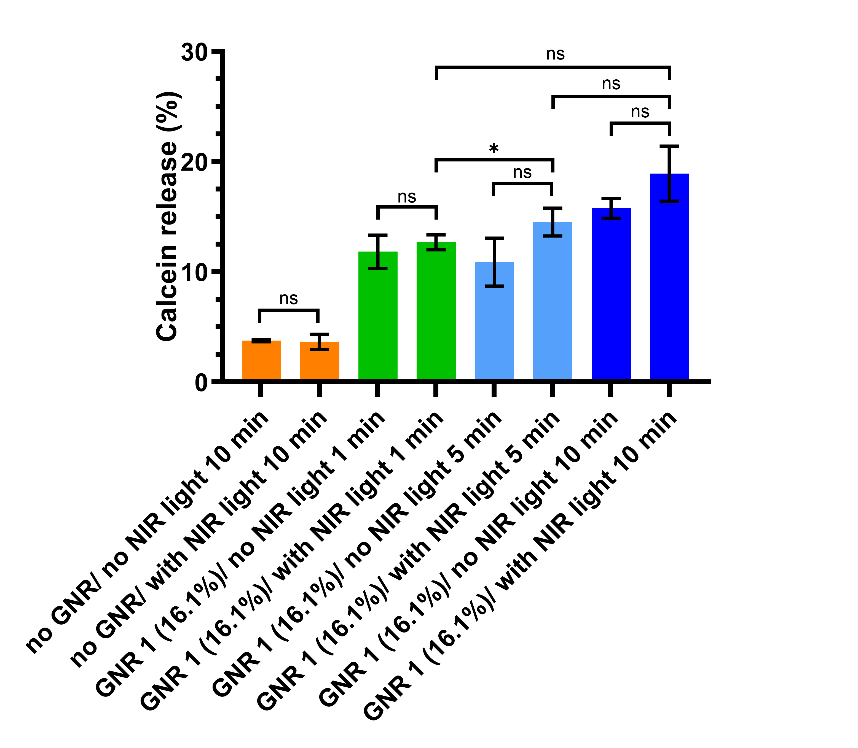
**

**Figure S7.** Time-dependent release efficiency of calcein encapsulated in GUVs with GNR 1 (16.1% Chol), N=3. Data are mean ± SD; N represent independent GUV preparations. At t = 0 (resuspension in fresh buffer after GNR incubation/purification), one sample was illuminated with NIR light and a matched control was kept w/o NIR illumination at room temperature. After a defined period (1, 5, or 10 min), both samples were processed in parallel to ensure consistent handling. Statistics: Tests and multiple-comparison handling are detailed in Experimental section, Statistical tests. Exact p-values: control (no GNRs), illuminated vs non-illuminated at 10 min: p = 0.783; GNR 1-functionalized sample, illuminated vs non-illuminated at 1 min: p = 0.451; at 5 min: p = 0.083; at 10 min: p = 0.208. Illuminated GNR 1-functionalized samples, 1 vs 5 min: p = 0.0246; 1 vs 10 min: p = 0.0690; 5 vs 10 min: p = 0.124 (see Table S2).

**
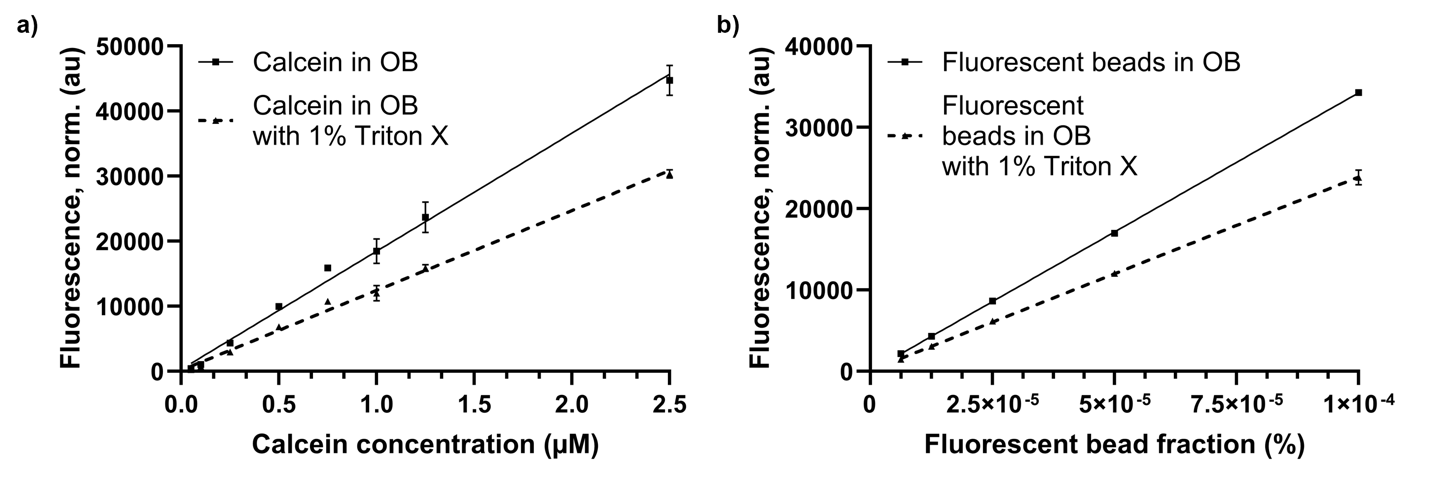
**

**Figure S8**. Calibration curve for calculating the concentration of: (a) calcein; (b) fluorescent beads released from GUVs with or without 1% Triton X, N=3. OB refers to the Outer Buffer of the GUVs.

**Table S2.** P-values for the cargo release experiments.

| **Samples compared (GUVs with calcein)** | **P value** |
| --- | --- |
| Un-functionalized GUVs with/without NIR illumination | 0.7238 |
| GNR 1 with/without NIR illumination | 0.2076 |
| GNR 2 with/without NIR illumination | 0.0003743 |
| GNR 3 with/without NIR illumination | 0.0005602 |
| GNR 4 with/without NIR illumination | 0.01104 |
| GNR 1 and GNR 2 with NIR illumination | 0.6063 |
| GNR 1 and GNR 3 with NIR illumination | 0.0006101 |
| GNR 1 and GNR 4 with NIR illumination | 0.3413 |
| GNR 2 and GNR 3 with NIR illumination | 4.661E-06 |
| GNR 2 and GNR 4 with NIR illumination | 0.07073 |
| GNR 3 and GNR 4 with NIR illumination | 0.0003885 |
| **Samples compared (GUVs with fluorescent beads)** | **P value** |
| Un-functionalized GUVs with/without NIR illumination | 7.206E-06 |
| GNR 3 with/without NIR illumination | 0.01443 |
| **Samples compared (GUVs with calcein, time-dependent release with GNR 1)** | **P value** |
| Un-functionalized GUVs with/without NIR illumination 10 min | 0.7833 |
| GNR 1 with/without NIR illumination 1 min | 0.4511 |
| GNR 1 with/without NIR illumination 5 min | 0.0832 |
| GNR 1 with/without NIR illumination 10 min | 0.2076 |
| GNR 1 with NIR illumination 1 and 5 min | 0.0246 |
| GNR 1 with NIR illumination 1 and 10 min | 0.06902 |
| GNR 1 with NIR illumination 5 and 10 min | 0.1242 |


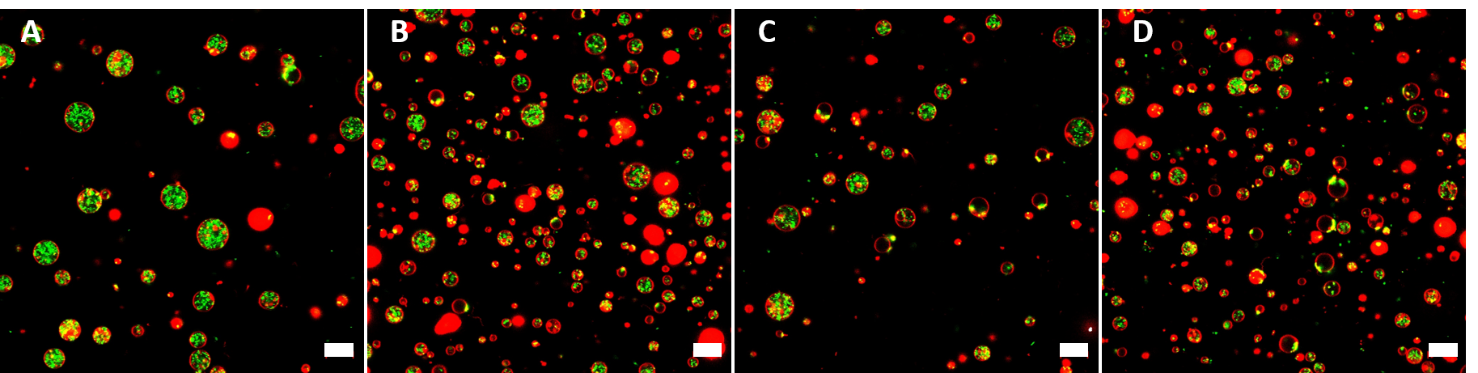


**Figure S9.** GUV encapsulating green fluorescent beads. A: GUVs without GNR, before NIR illumination; B: GUVs without GNR, after NIR illumination; C: GUVs with GNR, before NIR illumination; D: GUVs with GNR, after NIR illumination. The green signal comes from the green fluorescent beads, the red signal comes from the LissRhod PE lipid in the GUV membrane. GNRs are unlabeled. Scale bars are 10 µm.


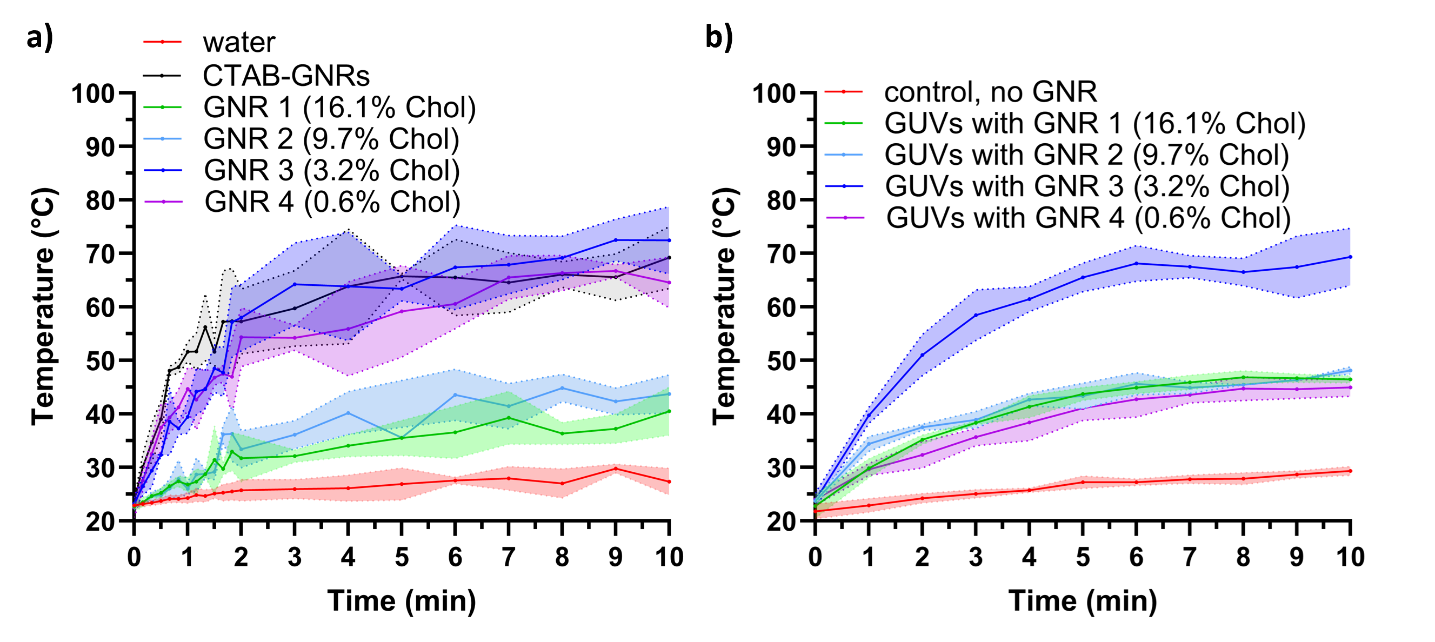


**Figure S10.** Temperature measurements of (a) NIR-illuminated pure CTAB-GNRs and cholesterol-functionalized GNRs, (b) NIR-illuminated GNR-functionalized GUVs. N=3.

**Release of Ampicillin and Killing of Bacteria**


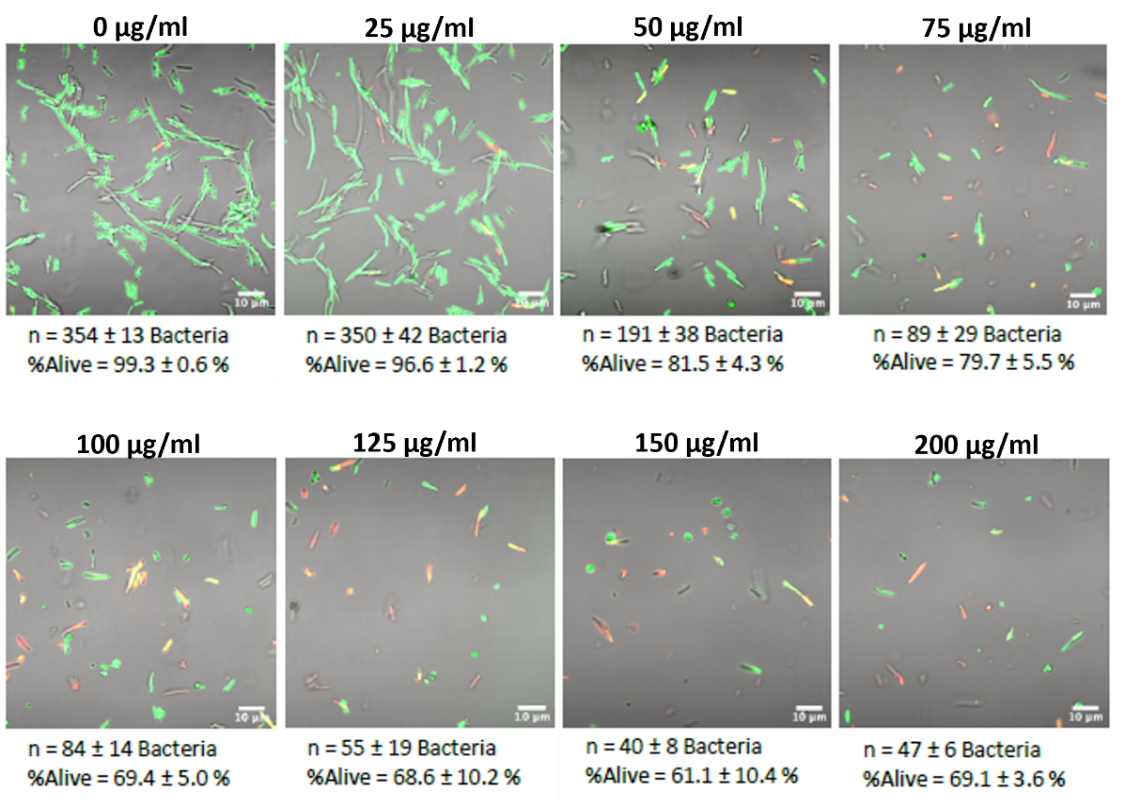


**Figure S11.** Bacterial viability testing in the presence of different concentrations of ampicillin**.** *E. coli* are grown for 20 minutes with different concentrations of ampicillin in the medium. The amount of live and dead bacteria was analyzed by Live/Dead staining. The mean was calculated from N=6 for each concentration. Scale bars are 10 µm.


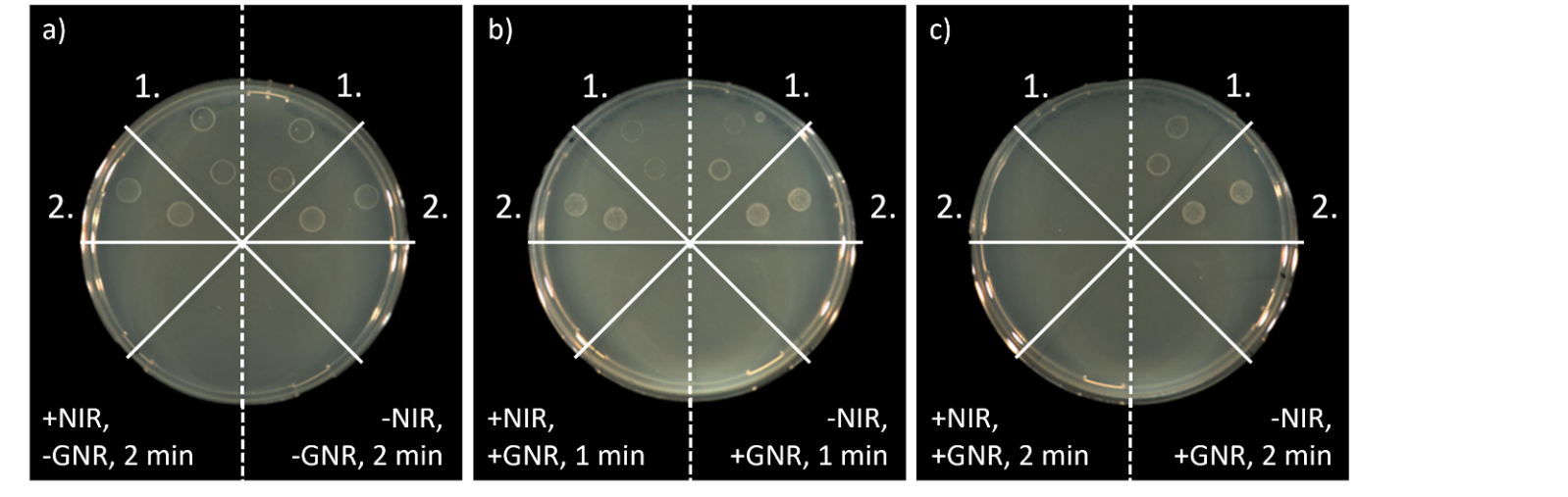


**Figure S12.** Bacterial viability following ampicillin release from (a) non-functionalized GUVs (without GNRs), (b) GNR-functionalized GUVs treated with 1 min of illumination, or (c) GNR-functionalized GUVs treated with 2 min of illumination. The left side of each image represents a sample after NIR light illumination, the right side is the control without NIR illumination; The numbers on the agar plate represent dilutions of the sample with LB medium: 1. undiluted sample, 2. 1:10 dilution.
